# Regeneration of the aged thymus by a single transcription factor

**DOI:** 10.1242/dev.103614

**Published:** 2014-04

**Authors:** Nicholas Bredenkamp, Craig S. Nowell, C. Clare Blackburn

**Affiliations:** Medical Research Council Centre for Regenerative Medicine, Institute for Stem Cell Research, School of Biological Sciences, University of Edinburgh, SCRM Building, 5 Little France Drive, Edinburgh EH16 4UU, UK

**Keywords:** Thymic involution, FOXN1, Organ regeneration, Mouse

## Abstract

Thymic involution is central to the decline in immune system function that occurs with age. By regenerating the thymus, it may therefore be possible to improve the ability of the aged immune system to respond to novel antigens. Recently, diminished expression of the thymic epithelial cell (TEC)-specific transcription factor Forkhead box N1 (FOXN1) has been implicated as a component of the mechanism regulating age-related involution. The effects of upregulating FOXN1 function in the aged thymus are, however, unknown. Here, we show that forced, TEC-specific upregulation of FOXN1 in the fully involuted thymus of aged mice results in robust thymus regeneration characterized by increased thymopoiesis and increased naive T cell output. We demonstrate that the regenerated organ closely resembles the juvenile thymus in terms of architecture and gene expression profile, and further show that this FOXN1-mediated regeneration stems from an enlarged TEC compartment, rebuilt from progenitor TECs. Collectively, our data establish that upregulation of a single transcription factor can substantially reverse age-related thymic involution, identifying FOXN1 as a specific target for improving thymus function and, thus, immune competence in patients. More widely, they demonstrate that organ regeneration in an aged mammal can be directed by manipulation of a single transcription factor, providing a provocative paradigm that may be of broad impact for regenerative biology.

## INTRODUCTION

The thymus, the obligate site of T lymphocyte development (Miller, 1961), is one of the first organs to degenerate in normal healthy individuals (Chinn et al., 2012). This process, termed ‘age-related thymic involution’, results in decreased production of naive T cells with age (Weng, 2006; Lynch et al., 2009; Chinn et al., 2012). This reduced output of naive T cells severely impairs the immune response to newly encountered antigens, making thymic involution a major cause of the age-related decline in immune system function. Thymic involution also results in an impaired capacity to recover adaptive immunity following immune depletion in patients (Holland and van den Brink, 2009; Lynch et al., 2009). The capacity to increase thymus function would therefore be beneficial in a wide variety of clinical settings, and consequently a number of strategies aimed at regenerating the aged thymus are currently under investigation (van den Brink et al., 2004; Holland and van den Brink, 2009; Markert et al., 2009).

The epithelial component of the thymic stroma is essential for intrathymic T cell development and undergoes a stereotypical age-related degeneration that is strongly implicated as a cause of age-related thymic involution (Mackall et al., 1998; Gray et al., 2006; Williams et al., 2008). The key transcription factor FOXN1 (Nehls et al., 1996) is crucially required throughout thymic epithelial cell (TEC) differentiation in the fetal and postnatal thymus (Chen et al., 2009; Nowell et al., 2011) and is downregulated with age in the thymic stroma (Ortman et al., 2002; Chen et al., 2009; Zook et al., 2011). Forced downregulation of *Foxn1* in the perinatal thymic epithelium results in loss of thymus homeostasis (Chen et al., 2009), while overexpression in young mice delays thymus degeneration (Zook et al., 2011). FOXN1 is thus implicated as one of the primary targets in age-related thymic involution.

Sex-steroid signaling is also thought to be an important regulator of involution. However, castration-induced thymic rebound was recently demonstrated to reflect enlargement of a thymus with an aged phenotype rather than restoration of the functionality and architecture of the young organ (Griffith et al., 2012). Therefore, the clinically important question of whether the effects of established age-related thymic involution can be reversed to drive rejuvenation of the fully involuted, aged thymus remains unanswered.

We have developed a novel transgenic model for conditional, inducible upregulation of FOXN1 function, and have used this to test the outcome of upregulating FOXN1 specifically in TECs in the fully involuted thymus. Our data establish that enhanced activity of this single transcription factor is sufficient to regenerate the fully involuted thymus such that its architecture, gene expression profile and function are restored to those characteristic of the juvenile organ. They further establish that this FOXN1-mediated thymic regeneration stems from proliferation of progenitor TECs.

## RESULTS

### A transgenic model for conditional inducible *Foxn1* expression

**Fig. 1.**
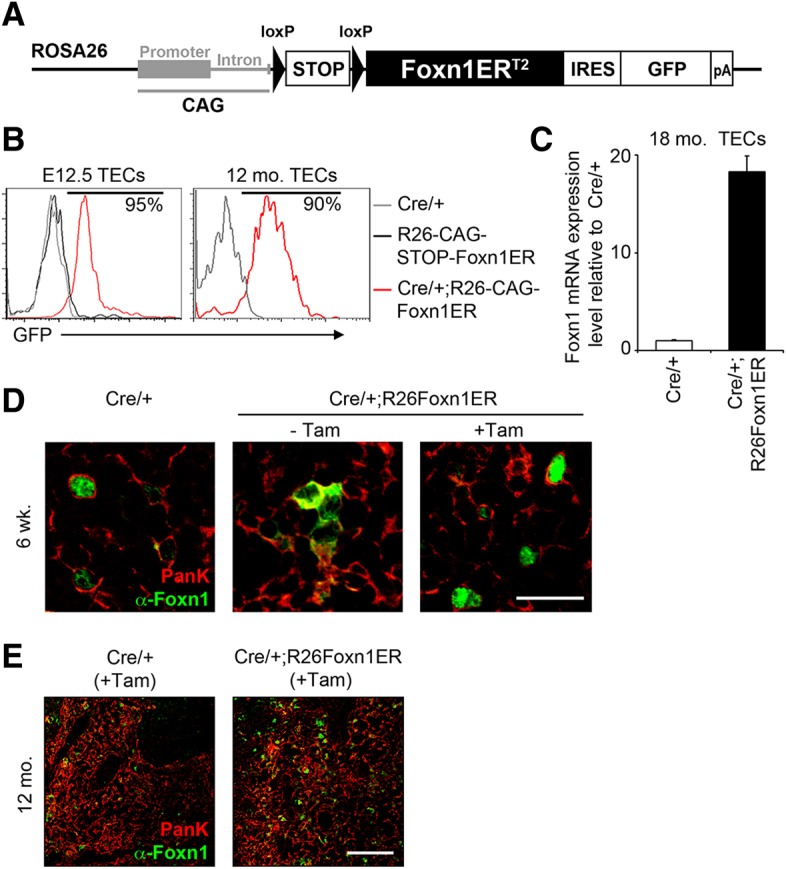
**Generation of a regulatable FOXN1 mouse model.** (A) Schematic of *Foxn1ER^T2^* transgene. (B) Plots show GFP expression reporting *CAG-Foxn1ER* in Cre/+, *ROSA26^CAG-STOP-Foxn1ERT2-IRES-GFP/+^* (STOP-Foxn1ER) and Cre/+;R26Foxn1ER TECs at the ages shown. (C) *Foxn1* expression in EpCAM^+^ TECs in 18-month-old Cre/+ and Cre/+;R26Foxn1ER mice, shown normalized to the geometric mean of three housekeeping (3HK) genes and relative to Cre/+. (D) Images show thymi from 6-week-old mice injected with carrier-only or 3 mg tamoxifen, at 2 days post-injection, after staining with the markers shown. Scale bar: 25 μm. (E) Images show thymi from tamoxifen-treated 12-month-old mice of the genotypes shown, after staining with the markers shown. Scale bar: 100 μm. Error bars, s.d. Mo, month. See also supplementary material Fig. S1. *n*≥3 for all experiments.

To test the hypothesis that upregulation of *Foxn1* in the aged, fully involuted thymus might reverse age-related thymic involution, we generated a transgenic mouse model that permits conditional, inducible overexpression of *Foxn1*, through production of a tamoxifen-inducible form of mouse FOXN1, FOXN1ER^T2^ (*ROSA26^CAG-STOP-Foxn1ERT2-IRES-GFP^* mice, referred to as R26Foxn1ER; Fig. 1A; supplementary material Figs S1 and S2). When induced by tamoxifen, this FOXN1ER^T2^ protein showed equivalent activity to native FOXN1 in a minimal responsive element reporter assay (supplementary material Fig. S3A) previously utilized to validate the activity of another FOXN1ER protein (Janes et al., 2004), and could induce expression of known FOXN1 targets to levels equivalent to native FOXN1 (supplementary material Fig. S3B). To activate expression of this transgene specifically in TECs we crossed the R26Foxn1ER line with *Foxn1^Cre/+^* (Cre/+) mice (Gordon et al., 2007), generating *ROSA26^CAG-Foxn1ERT2-IRES-GFP/+^*; *Foxn1^Cre/+^* (Cre/+;R26Foxn1ER) mice. Most, if not all, TECs in both fetal and adult Cre/+;R26Foxn1ER mice expressed *Foxn1ER* as reported by GFP expression (Fig. 1B), with *Foxn1ER* mRNA highly overexpressed in aged TECs (Fig. 1C). The tamoxifen inducibility of the FOXN1ER^T2^ fusion protein in TECs was confirmed by immunohistochemical (IHC) analysis (Fig. 1D,E) and immunoblotting (supplementary material Fig. S3C,D). In the absence of tamoxifen, no differences between control and Cre/+;R26Foxn1ER thymi were observed at any stage of development (shown for 12-month-old mice in supplementary material Fig. S4). Collectively, these data validated our experimental model.

### Increased FOXN1 function drives thymus regeneration in aged mice

**Fig. 2.**
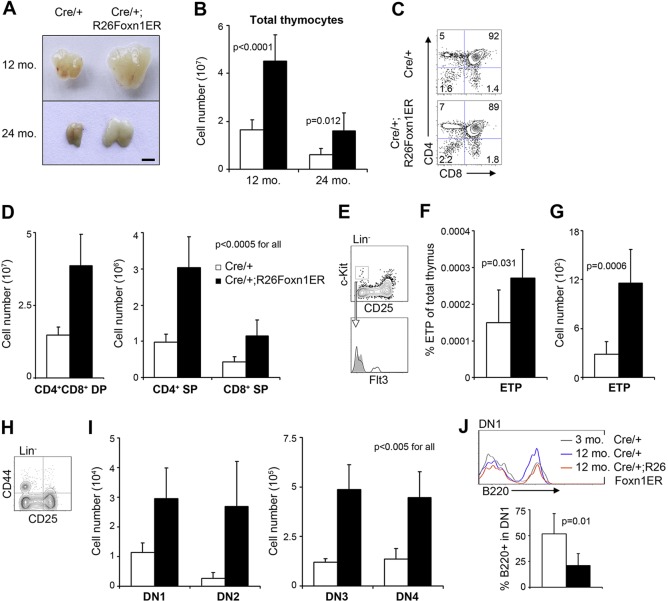
**Induction of FOXN1 drives thymus regeneration in aged mice.** Cre/+;R26Foxn1ER and Cre/+ mice (12 and 24 months old) were treated with tamoxifen for 1 month as described. (A) Representative thymic lobes. Scale bar: 2 mm. (B) Total thymocyte number (12 months, *n*=12; 24 months, *n*=6). (C) Plots show flow cytometric analysis of CD4 and CD8 staining on thymic cell preparations from 12-month-old mice of the genotypes shown after gating on CD45^+^ cells. (D) Numbers of CD4^+^CD8^+^ double positive (DP) and CD4^+^ and CD8^+^ single positive (SP) thymocytes in 12-month-old mice (*n*=10). Numerical fold change: DP, 2.6; CD4 SP, 3.1; CD8 SP, 2.6. (E) Gating strategy for ETPs (lin^−^ CD25^−^ Kit^+^ Flt3^+^) in 12-month-old mice (lin: CD4, CD8, CD3e, TCRβ, TCRγδ, NK1.1, CD11c, CD11b, Gr-1, Ter119, B220). Filled gray area, isotype control. (F) Frequency of ETPs as a percentage of total thymus cells (*n*=6). (G) Number of ETPs (*n*=6). (H) Analysis of double negative (DN) lin^−^ thymocytes using CD44 and CD25. Numerical fold change: 4.1. (I) Enumeration of DN populations (DN1, CD25^−^CD44^+^; DN2, CD25^+^CD44^+^; DN3, CD25^+^CD44^−^; DN4, CD25^−^CD44^−^; *n*=6). Numerical fold change: DN1, 2.6; DN2, 9.9; DN3, 4.1; DN4, 3.3. (J) B cell proportions in the DN1 compartment. Top panel, B220 staining after gating on DN1 cells (lin^−^CD25^−^CD44^+^, lin without B220). Bottom panel, average proportions of B220^+^ cells among DN1 (*n*=6). Black bars, Cre/+;R26Foxn1ER; white bars, Cre/+ controls, throughout. mo, month. Error bars, s.d.

To determine whether overexpression of FOXN1 in TECs could reverse established thymus involution in aged mice, we treated 12- and 24-month-old Cre/+;R26Foxn1ER mice with tamoxifen for 1 month to induce FOXN1ER^T2^ activity. Controls were provided by tamoxifen-treated Cre/+, and untreated Cre/+;R26Foxn1ER and Cre/+ littermates. These control groups revealed a mild and transient effect of tamoxifen treatment on thymocyte number, but no effects on thymic architecture or TEC subset distribution (supplementary material Fig. S4). Tamoxifen-induced upregulation of FOXN1 activity at both 12 and 24 months of age in Cre/+;R26Foxn1ER mice resulted in an overt increase in thymus size (Fig. 2A). Total thymocyte numbers increased more than 2.5-fold (Fig. 2B), with a proportional increase in the major thymocyte populations defined by expression of the CD4 and CD8 co-receptors (CD4^+^CD8^+^ double positive, CD4^+^ single positive, CD8^+^ single positive; Fig. 2C,D). The early thymic progenitor cell (ETP; lin^−^ CD25^−^ Kit^+^) population (Allman et al., 2003), which contains the most immature thymocytes, declines during age-related thymus involution (Min et al., 2006) and among ETPs, the Flt3^+^ fraction is considered to contain canonical intrathymic T cell progenitors (Adolfsson et al., 2005; Sambandam et al., 2005). This lin^−^ CD25^−^ Kit^+^ Flt3^+^ population was also significantly increased (Fig. 2E-G) and the CD4^−^CD8^−^ double negative (DN) thymocyte populations defined by CD44 and CD25 (also known as interleukin 2 receptor alpha chain, IL2ra) expression were expanded by greater than 2.5-fold relative to controls (Fig. 2H,I). Within the DN1 population (CD25^−^CD44^+^) the number of B cells (Lin^−^ B220^+^; B220 is also known as PTPRC) was reduced by half (Fig. 2J), suggesting increased commitment into the T cell lineage (Koch et al., 2008). These data establish that upregulation of FOXN1 activity is sufficient to promote increased thymic function in aged mice, as shown by expansion of all currently defined thymocyte populations, including ETPs.

We note that the increase in thymus size reported here, of 2.7-fold in 12-month-old mice, and 2.6-fold in 24-month-old mice after 1 month of tamoxifen treatment, corresponds well with those reported for castration-induced or keratinocyte growth factor (KGF)-induced rebound, which each typically result in a 2- to 3.5-fold increase in thymus size (Olsen et al., 2001; Alpdogan et al., 2006; Min et al., 2007; Williams et al., 2008; Griffith et al., 2012).

### FOXN1 upregulation restores the aged thymic epithelial microenvironment to a pre-involution phenotype

**Fig. 3.**
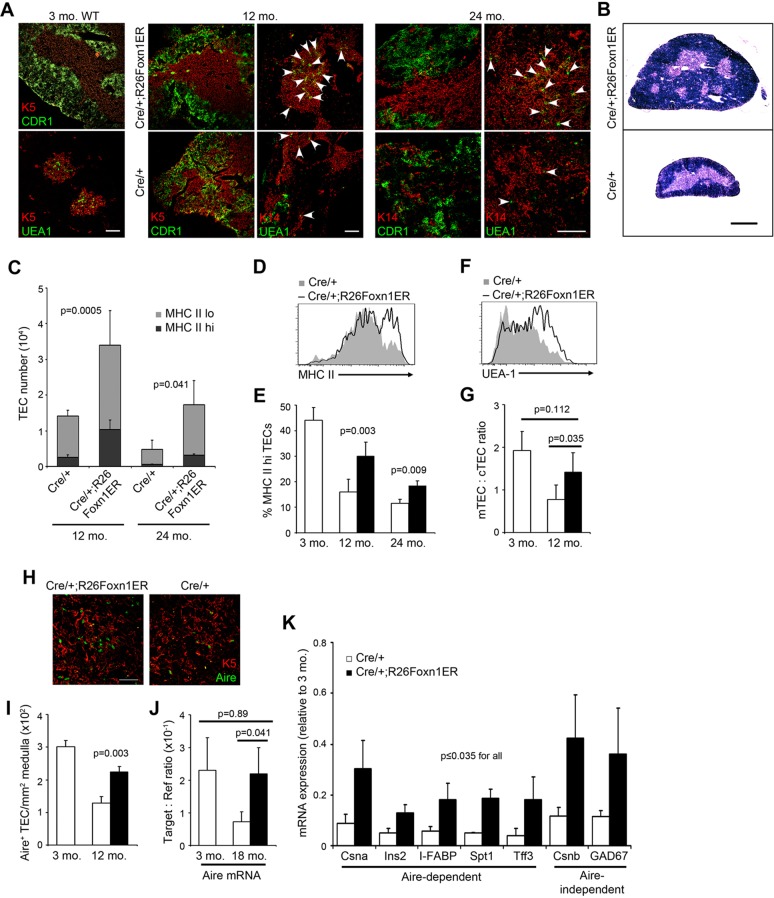
**Induction of FOXN1 restores the epithelial microenvironment in aged thymi.** Cre/+;R26Foxn1ER and littermate Cre/+ mice (12 and 24 months old) were treated with tamoxifen for 1 month; thymi from these mice were then microdissected and processed for analysis. (A) IHC for the markers shown, revealing cortical and medullary epithelial regions [cortex, CDR1; medulla, Keratin (K) 5 or 14 and UEA-1]. Arrowheads indicate UEA-1^+^ TEC clusters. Scale bars: 100 μm. (B) Hematoxylin and Eosin stained transverse sections from 12-month-old thymi. Number of medullary islets per lobe: Cre/+, 1.9; Cre/+;R26Foxn1ER, 3.6 (*n*=3, *P*=0.009). Scale bar: 1 mm. (C) TEC (CD45^−^EpCAM^+^) numbers, showing MHC Class II^hi^ (dark gray) and MHC Class II^lo^ (light gray) contributions (12 months, *n*=6; 24 months, *n*=3). Fold change in EpCAM^+^ TECs: 12 months, 2.4; 24 months, 3.6. (D) MHC Class II profiles of TECs from tamoxifen-treated 12-month-old mice of the genotypes shown. (E) MHC Class II^hi^ TECs as a proportion of total TECs (12 months, *n*=6; 24 months, *n*=3). Fold change in MHC Class II^hi^ TECs: 12 months old, 4.1; 24 months old, 5.7. (F) UEA-1 profile of TECs from 12-month-old tamoxifen-treated mice of the genotypes shown (UEA-1^+^ TECs are mTECs). (G) mTECs (UEA-1^+^, Ly51^−^) to cTECs (UEA-1^−^, Ly51^+^) ratio in 3- and 12-month-old mice, as determined from flow cytometric analysis (12 months, *n*=5). (H) IHC of tamoxifen-treated 12-month-old thymi of the genotypes shown, showing AIRE staining in mTECs (K5 positive). Scale bar: 50 μm. (I) Number of AIRE^+^ cells per mm^2^ of medullary area in tamoxifen-treated 3- and 12-month-old mice (12 months, *n*=3). (J) RT-qPCR analysis of *Aire* expression in mTECs from 3- and 18- to 24-month-old tamoxifen-treated mice, normalized to the geometric mean of three housekeeping genes (*n*=3). (K) TRA expression in total TECs in 12-month-old mice, shown relative to young TECs after normalization to the TEC housekeeping gene *Eva* (*Mpzl2*) (expression level in TECs at 3 months=1; *n*≥3 for all genes). mo, month; MHC II, MHC Class II. Error bars, s.d.

Age-related thymic involution is defined by a stereotypical deterioration of the thymic epithelial compartment, characterized by reduced distinction between the cortical and medullary regions; reduction in the number of medullary islets per thymic lobe (Griffith et al., 2012); a decline in total TEC number and in the rate of TEC turnover (Gray et al., 2006); a specific loss of some TEC subsets (Chen et al., 2009), including a proportional decrease in MHC Class II^hi^ TECs; and reduced expression of tissue-restricted antigens (Griffith et al., 2012). Therefore, we tested the effect of FOXN1 upregulation on these parameters. In striking contrast to aged-matched controls, the stromal compartment in 12- and 24-month-old tamoxifen-treated Cre/+;R26Foxn1ER thymi showed clearly defined cortical and medullary regions, establishing that upregulation of FOXN1 activity restored thymic epithelial architecture to be near indistinguishable from juvenile controls (Fig. 3A). Thymi from tamoxifen-treated 12-month-old Cre/+;R26Foxn1ER mice also exhibited increased numbers of medullary islets compared with age-matched controls, similar to juvenile thymi (Fig. 3B) (Griffith et al., 2012). The number of EpCAM^+^ TECs (Fig. 3C) and both number and proportion of MHC Class II^hi^ TEC were significantly increased (Fig. 3C-E). Furthermore, the ratio of medullary TECs (mTECs) to cortical TECs (cTECs), which decreases with age (Gray et al., 2006; Chen et al., 2009), was restored to near that of a young thymus (Fig. 3F,G).

We also examined TEC subset distribution. The *Ulex europaeus* agglutinin-1 (UEA-1)^hi^ mTEC subpopulation, which is proposed to regulate medullary organization (Naquet et al., 1999), declines with age and is also preferentially lost following premature downregulation of *Foxn1* expression in the postnatal thymus (Chen et al., 2009). This phenotype was rescued by upregulation of FOXN1 in aged thymi; the relative numbers of UEA-1^hi^ mTEC in tamoxifen-treated Cre/+;R26Foxn1ER mice were similar to those of young wild-type controls (Fig. 3A). The autoimmune regulator AIRE plays a crucial role in negative selection of thymocytes as it regulates expression in TECs of a set of genes that are otherwise expressed only in specific tissues or cell types (tissue-restricted antigens or TRAs) (Anderson et al., 2002; Mathis and Benoist, 2009). AIRE is expressed in an mTEC subpopulation that declines numerically with age and AIRE^+^ mTEC number has been linked to *Foxn1* expression levels (Nowell et al., 2011). The number of AIRE^+^ mTECs and the expression of *Aire* mRNA was significantly increased in aged, tamoxifen-treated Cre/+;R26Foxn1ER thymi versus controls (Fig. 3H-J; see supplementary material Fig. S5 for gating strategy for flow cytometric analysis and sorting of TEC subsets, and validation). The AIRE-dependent TRAs casein alpha (*Csna*; *Csn1s1* – Mouse Genome Informatics), insulin 2 (*Ins2*), intestinal fatty acid binding protein (*I-FABP*; *Fabp2* – Mouse Genome Informatics), salivary protein 1 (*Spt1*) and trefoil factor 3 (*Tff3*), which decrease during thymus involution (Anderson et al., 2002; Derbinski et al., 2005), were also upregulated upon induction of FOXN1 activity (Fig. 3K), establishing the functionality of the new AIRE^+^ mTECs. The two AIRE-independent TRAs tested, casein beta (*Csnb*; *Csn2* – Mouse Genome Informatics) and glutamate decarboxylase 67 (*GAD67*; *Gad1* – Mouse Genome Informatics) (Anderson et al., 2002), were also upregulated. Collectively, these data indicate that thymus structure and function were regenerated to closely resemble their pre-involution phenotypes in the FOXN1-regenerated aged thymus.

### FOXN1 upregulation restores the expression of genes required for TEC function

**Fig. 4.**
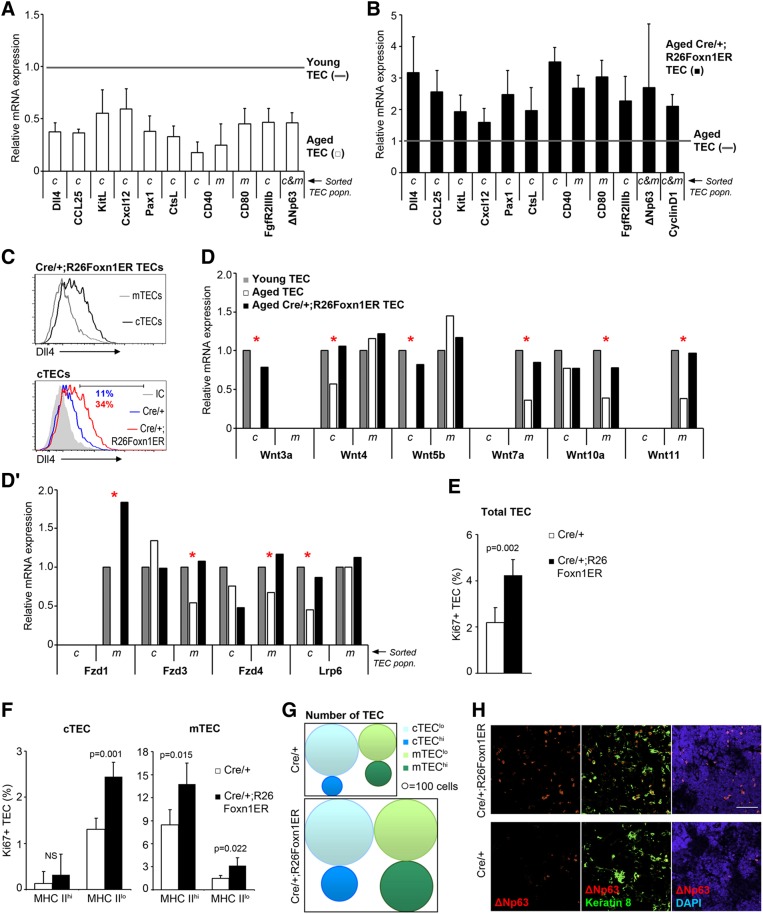
**Induction of FOXN1 elevates epithelial proliferation and gene expression.** (A) mRNA expression levels of the genes shown in mTECs (m) and/or cTECs (c) from young (3­-month-old) and aged (18-month-old) wild-type mice, shown as fold-change relative to young mice after normalization to the geometric mean of three housekeeping (3HK) genes (3 months, *n*=1; 18 months, *n*=3). (B) mRNA expression levels in TECs from tamoxifen-treated 18- to 24-month-old mice of the genotypes shown, shown as fold change relative to Cre/+ controls (gray line) after normalization to the geometric mean of 3HK genes (*n*=3). (C) Top: DLL4 expression on cTECs and mTECs from tamoxifen-treated 12-month-old Cre/+;R26Foxn1ER mice. Bottom: DLL4 expression on cTECs from tamoxifen-treated 12-month-old Cre/+ and Cre/+;R26Foxn1ER mice. (D,D′) mRNA expression levels of the genes shown in mTECs (m) and cTECs (c) from tamoxifen-treated young (3-month-old) and aged (12-month-old) mice, shown as fold-change relative to cTECs or mTECs from young mice after normalization to the geometric mean of 3HK. Data represent the expression levels in TECs pooled from four mice. Red asterisks indicate genes downregulated in involution and restored upon FOXN1 upregulation. (E,F) Proportion of Ki67^+^ TECs in total EpCam^+^ TECs (E) and the major TEC subpopulations (F) (mTECs: UEA-1^+^, Ly51^−^; cTECs: UEA-1^−^, Ly51^+^) from tamoxifen-treated 12-month-old mice of the genotypes shown (*n*=4). (G) Schematic of TEC subpopulation numbers in tamoxifen-treated 12-month-old mice of the genotypes shown. hi and lo refer to MHC Class II surface expression levels. (H) Analysis of tamoxifen-treated 12-month-old thymi showing ΔNp63 staining. Scale bar: 50 μm. Black bars, Cre/+;R26Foxn1ER; white bars, Cre/+ controls, throughout. c, cTECs; m, mTECs; mo, month; MHC II, MHC Class II. Error bars, s.d.

To probe further the effects of FOXN1 upregulation on the thymic microenvironment, we next tested the effects of normal ageing and of FOXN1 induction on a panel of genes required cell-autonomously in TECs for TEC development or for T cell differentiation and repertoire selection. Delta-like 4 (*Dll4*), chemokine (C-C motif) ligand 25 (*Ccl25*), kit ligand (*Kitl*) and chemokine (C-X-C motif) ligand 12 (*Cxcl12*) are putative transcriptional targets of FOXN1 that are expressed in cTECs and are required for the early stages of T cell development (Rodewald et al., 1997; Koch et al., 2008; Trampont et al., 2010; Zlotoff et al., 2010; Nowell et al., 2011; Calderon and Boehm, 2012); *Cd40*, *Cd80*, cathepsin L (*Ctsl*) and paired box 1 (*Pax1*) are expressed specifically in mTECs (*Cd80*), cTECs (*Ctsl*, *Pax1*) or both (*Cd40*), and are required in TECs to mediate normal T cell development (Reiser and Schneeberger, 1994; Van Den Berg et al., 1996; Wallin et al., 1996; Honey et al., 2002; Akiyama et al., 2008). Expression of all of these genes was substantially downregulated in aged versus young cTECs (Fig. 4A; see supplementary material Fig. S5 for gating strategy for flow cytometric analysis and sorting of TEC subsets, and validation), consistent with an age-related decline in TEC functionality. Fibroblast growth factor receptor 2 isoform IIIb (*FgfR2IIIb*), the receptor for fibroblast growth factors 7 and 10 (Revest et al., 2001), and the delta N isoform of transformation-related protein 63 (p63, also known as TRP63) ΔNp63 (Candi et al., 2007; Senoo et al., 2007) were also significantly downregulated in aged versus young TECs (Fig. 4A; note that *FgfR2IIIb* expression is restricted to cTECs), consistent with decreased TEC proliferation with age (Gray et al., 2006).

Tamoxifen-treated Cre/+;R26Foxn1ER mice exhibited increased expression of *Dll4* in TECs, such that the relative expression level was similar to that in TECs from 3-month-old mice (Fig. 4B,C; supplementary material Fig. S5 and Fig. S6A). Strikingly, *Dll4* was upregulated only in cTECs (Fig. 4C), where it was predominantly expressed in the MHC Class II^hi^ subset (supplementary material Fig. S6A). Thus, although functional FOXN1 was upregulated in all TECs, FOXN1 targets maintained their expected TEC subset-specific expression. Of note is that the expression profile for DLL4 protein on TECs in the FOXN1-regenerated thymus is characteristic of the young rather than the aged thymus (Fiorini et al., 2008; Koch et al., 2008). *Ccl25*, *Kitl*, *Cxcl12*, *Cd40*, *Cd80*, *Ctsl* and *Pax1* were also upregulated close to levels found in 3-month-old TECs (Fig. 4B). Increased expression of these genes in the aged thymus in response to FOXN1 upregulation provides a mechanistic explanation for the specific impacts observed on the early thymocyte compartment, including the proportional drop in intrathymic B cells which probably results from increased DLL4 expression (Hozumi et al., 2008; Koch et al., 2008).

Among signaling pathways, the Wnt pathway is reportedly most affected in TECs by age-related thymic involution (Griffith et al., 2012). Our analysis confirmed substantially downregulated expression of many Wnt pathway genes in aged versus young TECs, and extended previous findings to show that many of these genes are differentially regulated in c- and mTECs (Fig. 4D,D′). Strikingly, expression of most of the Wnt pathway genes analyzed were restored to young levels in tamoxifen-treated aged Cre/+;R26Foxn1ER TECs (Fig. 4D,D′). Again, these genes maintained their normal TEC subset-specific expression upon FOXN1 upregulation. Of note is that lymphotoxin receptor β (*Ltbr*), interleukin 7 (*Il7*), v-rel avian reticuloendotheliosis viral oncogene homolog B (*Relb*) and intracellular adhesion molecule 1 (*Icam1*) were expressed at equivalent levels in aged, young and tamoxifen-treated aged Cre/+;R26Foxn1ER TECs (supplementary material Fig. S7A).

Collectively, these data establish that reduced FOXN1 expression is the crucial factor limiting expression of a wide range of genes in aged TECs, and provide additional evidence that the FOXN1-regenerated thymus very closely resembles the young thymus.

### FOXN1 mediates thymus regeneration by increasing proliferation of progenitor TEC

TEC proliferation is known to decrease with age, with an approximately threefold decrease in the proportion of proliferating TECs from 4 weeks to 10 months of age (Gray et al., 2006), and a link between FOXN1 and TEC proliferation has been established in fetal thymus (Itoi et al., 2001). To determine the cellular mechanism through which FOXN1 upregulation leads to thymus regeneration, we therefore tested whether the increase in TEC numbers resulted from increased TEC proliferation, using Ki67 staining. The proportion of proliferating cells in the total TEC population increased by twofold upon upregulation of FOXN1 function in 12-month-old mice (Fig. 4E; supplementary material Fig. S6B). Among cTECs, proliferation was significantly increased only in the MHC Class II^lo^ compartment, which contains putative progenitor cTECs (Fig. 4F). The numbers of both MHC Class II^lo^ and MHC Class II^hi^ cTECs increased, however, indicating that in the regenerated thymi this MHC Class II^hi^ cTEC compartment was rebuilt from a progenitor TEC pool (Fig. 4F,G). We found increased proliferation of both MHC Class II^lo^ and MHC Class II^hi^ mTECs (Fig. 4F); both of these mTEC compartments contain proliferating cells in young mice (Tykocinski et al., 2008).

To investigate the molecular basis of this increased proliferation, we analyzed expression of genes with roles in cell cycle progression and TEC proliferation. We observed an increase in the number of TECs expressing ΔNp63, which marks proliferative potential in epithelial cells including TECs (Candi et al., 2007; Senoo et al., 2007), in tamoxifen-treated aged Cre/+;R26Foxn1ER thymi versus age-matched controls (Fig. 4A,B,H); expression of the cell cycle regulator cyclin D1 and of *Fgf2RIIIb* was also upregulated (Fig. 4A,B). *Fgf2RIIIb* was previously been reported to be regulated by FOXN1 (Tsai et al., 2003). However, neither *Ccnd1* nor *p63* has previously been suggested to be a direct or indirect transcriptional target of FOXN1, and these data therefore expand the set of FOXN1-regulated targets to include these genes. As ΔNp63, cyclin D1 and *Fgf2RIIIb* have all previously been shown to regulate TEC proliferation (Robles et al., 1996; Revest et al., 2001), this provides a mechanistic link between increased FOXN1 expression and the expansion of TECs observed in these experiments.

Of note is that we could not detect interleukin 22 receptor (*Il22r*) expression (Dudakov et al., 2012) in tamoxifen-treated Cre/+;Foxn1ER TEC in aged mice (supplementary material Fig. S7B), indicating that FOXN1-mediated thymus regeneration acts via an IL22-independent mechanism.

### FOXN1-mediated thymus regeneration impacts the peripheral immune system by increasing naive T cell numbers

**Fig. 5.**
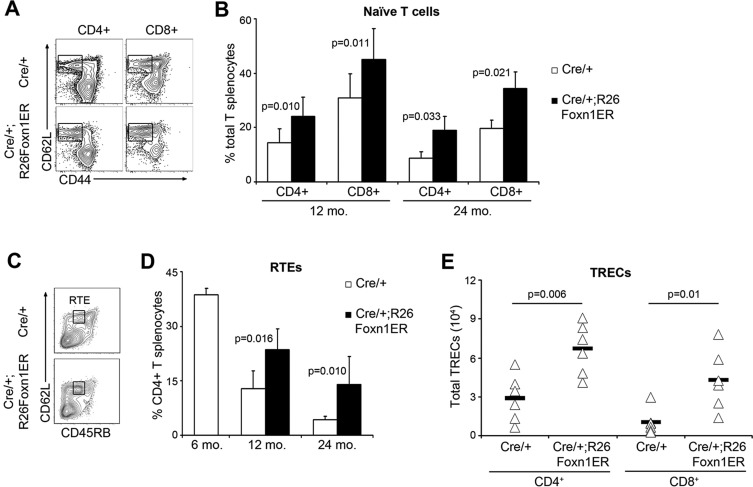
**Induction of FOXN1 improves thymic output in aged thymi.** (A) Splenic cell preparations from tamoxifen-treated 12-month-old mice of the genotypes shown, after gating on CD3^+^ cells. Naive T cells are CD62L^+^CD44^lo^. (B) Average proportions of CD4^+^ and CD8^+^ naive T cells in tamoxifen-treated 12- and 24-month-old mice of the genotypes shown (12 months, *n*=6; 24 months, *n*=3). (C) Splenic cell preparations from tamoxifen-treated 24-month-old mice of the genotypes shown, after gating on CD3^+^CD4^+^ cells. Recent thymic emigrants (RTEs) are CD62L^+^CD45RB^int^. (D) Average proportions of RTEs in tamoxifen-treated 12- and 24-month-old mice (12 months, Cre/+ *n*=4, Cre/+;R26Foxn1ER *n*=6; 24 months, Cre/+ *n*=6, Cre/+;R26Foxn1ER *n*=8). (E) Number of T cell receptor excision circles (TRECs) in naive splenic T cells (CD62L^+^CD45RB^+^) from tamoxifen-treated 24-month-old mice (*n*=6). Black bars, Cre/+;R26Foxn1ER; white bars, Cre/+ controls, throughout. mo, month. Error bars, s.d.

A crucial question is whether these changes in thymocyte numbers translate into an altered peripheral lymphocyte pool. Age-related thymic involution leads to a numerical and proportional decrease in naive T cells in the peripheral pool, that is thought to be a major factor in the capacity of the immune system to respond to new antigenic challenge (Goronzy and Weyand, 2005). Both CD4^+^ and CD8^+^ splenocytes from aged, tamoxifen-treated Cre/+;R26Foxn1ER mice contained a significantly higher proportion of CD3^+^CD62L^+^CD44^lo^ (CD62L is also known as SELL) naive T cells (Budd et al., 1987) than controls (Fig. 5A,B), demonstrating improved export of naive T cells from the thymus. To specifically identify recent thymic emigrants (RTEs), the CD45RB phenotype of CD4^+^ splenocytes was analyzed (Boursalian et al., 2004). Upregulation of FOXN1-activity in 24-month-old Cre/+;R26Foxn1ER mice resulted in a higher proportion of CD45RB^lo^ RTEs among naive splenic T cells (supplementary material Fig. S8A,B), and the proportion and numbers of RTEs in the total splenic CD4^+^ compartment of 12- and 24-month-old tamoxifen-treated Cre/+;R26Foxn1ER mice were increased by twofold versus controls by 1 month after treatment commenced (Fig. 5C,D; supplementary material Fig. S8B). The increase in RTEs was also evidenced by a greater than twofold increase in T cell receptor excision (TREC) (Sempowski et al., 2002) number in both CD4^+^ and CD8^+^ CD45RB^+^ naive splenocytes (Fig. 5E; supplementary material Fig. S8C). Thus, upregulation of FOXN1 in aged thymi resulted in an increase in the export of RTEs to the periphery with consequent increase in the numbers and proportions of naive T cells in the peripheral immune system.

## DISCUSSION

We have shown that upregulation of a crucial regulator of TEC differentiation, FOXN1, is sufficient to drive regeneration of the aged thymus such that it closely resembles the juvenile, pre-involution thymus in terms of architecture, gene expression and functionality. This FOXN1-mediated thymus regeneration results in improved thymic output in aged mice, with increased numbers of naive T cells exported to peripheral lymphoid organs. Collectively, our data demonstrate that the major impacts of age-related involution on thymus structure and function can be substantially reversed by restoring FOXN1 function, establishing that loss of FOXN1 expression is the principal cause of these components of thymic involution. They further provide insight into the mechanisms by which FOXN1 mediates thymus regeneration.

Our findings contrast with the outcome of provoking thymus regeneration via sex-steroid ablation, which a recent study has shown results in transient generation of an enlarged, but phenotypically old thymus with no change in TRA expression or medullary complexity, and no upregulation of key regulatory and functional genes compared with aged controls (Griffith et al., 2012). In addition, most of the genes involved in the Wnt signaling pathway that were examined herein were downregulated during age-related thymic involution, in agreement with a previous study (Griffith et al., 2012). These genes were restored to young expression levels upon upregulation of FOXN1 activity in aged TECs, whereas their expression is not restored following sex steroid ablation-mediated rebound (Griffith et al., 2012). *In vivo* manipulation of FOXN1 expression, which not only enlarges the aged thymus but also restores its function, therefore presents a promising alternative strategy for clinical thymus regeneration – for instance by small molecule manipulation of signaling pathways that regulate FOXN1 expression or directly augment FOXN1 function.

FOXN1 is a powerful regulator of TEC differentiation (Blackburn et al., 1996; Nehls et al., 1996; Chen et al., 2009; Nowell et al., 2011), is required throughout TEC development from exit from the earliest progenitor TEC state to terminal differentiation in both cTEC and mTEC sub-lineages (Nowell et al., 2011), and has also been implicated as a regulator of proliferation of fetal TECs (Itoi et al., 2001). In the context of our experimental model, however, upregulation of FOXN1 did not drive all TECs in the aged thymus to terminal differentiation, but rather caused a sustained increase in the size of both progenitor and terminally differentiated TEC compartments, driven by proliferation of progenitor cells. This increased proliferation was accompanied by upregulation of genes that promote cell cycle progression (cyclin D1, *ΔNp63*, *FgfR2IIIb*) and that are required in TECs to promote specific aspects of T cell development (*Dll4*, *Kitl*, *Ccl25*, *Cxcl12*, *Cd40*, *Cd80*, *Ctsl*, *Pax1*). These data suggest a mechanistic basis for cell cycle promotion by FOXN1, and indicate that factors in addition to FOXN1 must limit the onset of terminal differentiation in progenitor TECs. They further establish, to our knowledge for the first time, that cyclin D1 and ΔNp63 are direct or indirect targets of FOXN1. In conjunction with a recent report demonstrating that the retinoblastoma family of proteins can negatively regulate *Foxn1* expression in TECs (Garfin et al., 2013), they thus suggest a complex interplay between FOXN1 expression and cell cycle regulation.

Recently, Sun and colleagues reported that delivery of a FOXN1-expression plasmid to the aged thymus by intrathymic injection resulted in increased thymus size. However, as cellular composition, architecture and function of the thymus were not examined, that study was not informative regarding the effect of FOXN1 upregulation on hallmarks of thymic involution, including altered TEC phenotype and function (Sun et al., 2010). The importance of evaluating these parameters is highlighted by the findings of Griffiths and colleagues, discussed above, that the degenerative changes characteristic of age-associated thymic involution persist despite increased thymic size during castration-induced thymic rebound (Griffith et al., 2012). In a separate study, Zook and colleagues reported that overexpression of FOXN1 from the human keratin 14 (hK14; *KRT14* – Human Gene Nomenclature Database) promoter, which can drive broad expression in fetal and postnatal TECs including cTECs (Laufer et al., 1996), delays but does not prevent the onset of age-related thymus involution (Zook et al., 2011). As thymic involution still occurred in the presence of FOXN1 overexpression in this model (Zook et al., 2011), these data suggested that upregulation of FOXN1 should not be sufficient to regenerate the age-related thymus. In our experiments, increased FOXN1 expression restored thymic architecture and function, despite the fact that involution was fully established before FOXN1 function was upregulated. Our data thus demonstrate unequivocally that FOXN1 upregulation is sufficient to reverse the crucial hallmarks of age-related thymic involution, based on all parameters commonly used to measure thymus structure and function. The reversibility of these hallmark changes indicates that the involuted state induced in the thymus by ageing is not locked, for instance by epigenetic or other mechanisms that result in permanent loss of key functions, even once fully established. Our data thus suggest that in the ageing and aged thymus, FOXN1 or its upstream regulators must be continuously repressed by an active mechanism in order to suppress FOXN1-mediated regeneration.

Since the discovery of induced pluripotent stem cells (Takahashi and Yamanaka, 2006), interest has increased in the capacity of a single or a few transcription factors to program/reprogram cell fate – for instance, via direct conversion of cell fates or by directing differentiation of embryonic stem cells into defined cell types (Weintraub et al., 1989; Seguin et al., 2008; Zhou et al., 2008; Graf and Enver, 2009; Antonica et al., 2012). Our findings demonstrate that in the aged thymus FOXN1 is sufficient to promote proliferation of progenitor TECs and differentiation of these cells into fully functional thymic epithelium, extending this approach to the important goal of whole organ regeneration *in vivo*. As discussed, our data show that upregulating FOXN1 function leads to upregulation of key genes required for TEC proliferation and function. An important emerging question is therefore whether FOXN1 regulates this diverse set of targets via classical transactivation, or functions either additionally or alternatively as a ‘pioneer factor’, as recently demonstrated for FoxA proteins (Cirillo et al., 2002; Zaret and Carroll, 2011).

Collectively, the data presented herein have identified both the cell type and the gene required for thymus regeneration. These findings highlight FOXN1 as a specific target for strategies aiming to achieve thymus regeneration in patients, underlining the need to understand both its transcriptional regulation and downstream targets. Finally, by establishing that modulation of a single transcription factor is sufficient to instigate regeneration of an entire organ, our findings provide a provocative paradigm that may be of broad impact for regenerative biology strategies.

## MATERIALS AND METHODS

### Ethics statement

All animal work was conducted according to UK Home Office guidelines, as established in the Animals (Scientific Procedures) Act 1986.

### Mice

*Rosa26^CAG-STOP-Foxn1ERT2-IRES-GFP/+^* (R26Foxn1ER) mice were backcrossed onto C57BL/6 for at least three generations then maintained via intercrossing. *Foxn1^Cre/+^* mice (Gordon et al., 2007) were maintained as homozygotes and crossed with *Rosa26^CAG-STOP-Foxn1ERT2-IRES-GFP/+^* mice as described to generate *Foxn1^Cre/+^*;*Rosa26^CAG-Foxn1ERT2-IRES-GFP/+^* (Cre/+;R26Foxn1ER mice). Littermate controls were used in each experiment. For timed matings, noon of the day of the vaginal plug was taken as E0.5.

### Generation of the Foxn1ER^T2^ targeting vector

A construct containing the targeting cassette (Fig. 1A; supplementary material Fig. S1A) was generated by standard molecular biology techniques (Nowell et al., 2011) and verified by sequencing. Conventional subcloning was used for all cloning steps.

### Southern blotting

Genomic DNA was processed for Southern blotting as described (Morris et al., 2006; Nowell et al., 2011).

### Gene targeting and blastocyst injection

Mouse sv129/ola ES cells (line E14tg2a) were electroporated with linearized targeting vector (supplementary material Fig. S1A) and grown under Geneticin selection. Correctly targeted clones were identified by Southern analysis (supplementary material Fig. S1B), expanded and injected into C57BL/6 blastocysts to generate chimeric mice. Germ-line transmission was confirmed for two independently targeted cell clones (supplementary material Fig. S1B); the resulting mouse line was designated *ROSA26^CAG-STOP-Foxn1ERT2-IRES-GFP^* (called R26Foxn1ER herein). The neomycin resistance cassette was removed by crossing of founder R26Foxn1ER mice with *Tg(CAG-FLPe)* mice (Wallace et al., 2007) and confirmed by Southern analysis (supplementary material Fig. S1C).

### Transient transfection

A luciferase reporter cell system (Janes et al., 2004) containing the wild-type and mutated minimal FOXN1 response elements (Schlake et al., 1997) was transiently transfected with empty, *CAG-Foxn1* and *CAG-Foxn1ER^T2^* vectors using Lipofectamine 2000 (Invitrogen). Cells were cultured for 48 h with or without 4-hydroxytamoxifen (1 μM, Sigma), then assayed using a Luciferase Assay System (Promega) and analyzed on a Mediators PhL Luminometer (ImmTech).

### Tamoxifen treatment

Mice were treated with a single intraperitoneal (IP) injection of tamoxifen (Sigma) prepared in ethanol and diluted appropriately in Cremophor (Sigma)/PBS. For regeneration experiments, mice were treated with a single 3 mg IP injection of tamoxifen and then with tamoxifen citrate salt (Sigma) in the drinking water (0.05 mg/ml) for one month.

### Antibodies

The antibodies used for IHC and flow cytometry are listed in supplementary material Table S1.

### Flow cytometry

#### Analysis

Mouse fetal TECs were processed for flow cytometric analysis as described (Bennett et al., 2002; Nowell et al., 2011); adult TECs were enzymatically digested to single cell suspensions as described (Gray et al., 2008) and processed for cytometric analysis without further enrichment. Naive T cells (CD3^+^CD62L^+^CD44^lo^) (Budd et al., 1987) and RTEs (CD3^+^CD4^+^CD62L^+^CD45RB^int^) (Boursalian et al., 2004) were identified among splenocytes as described. Data were acquired using LSR Fortessa (BD Biosciences) and analyzed using FlowJo software (Tree Star).

#### Sorting

For isolation of adult TECs, TECs were enzymatically digested to single cell suspensions and enriched as described (Gray et al., 2008). Total TECs (EpCAM^+^) or cTECs (EpCAM^+^Ly51^+^) or mTECs (EpCAM^+^Ly51^−^) were purified after gating against CD45^−^Ter119^−^ cells. For isolation of splenocytes, mouse spleens were mechanically disrupted and red blood cells were lysed. CD4^+^ and CD8^+^ naive splenocytes were purified after gating on CD3^+^CD62L^+^CD45RB^+^ cells. Cell sorting was performed using a FACSAriaII (BD Biosciences).

### Immunohistochemistry

Adult thymi were processed for IHC as described (Nowell et al., 2011). Isotype controls (not shown) were included in all experiments. Staining was analyzed using a Leica AOBS confocal microscope (Leica Microsystems). The images presented are either single optical sections or projected focus stacks of serial optical sections. The number of FOXN1^+^ or AIRE^+^ TECs per area was established by analyzing three areas on each of two non-sequential sections from each of three individual mice. Statistical analysis was performed on the average number of FOXN1^+^ or AIRE^+^ cells per condition.

### RNA isolation

RNA was prepared using an RNAeasy Mini Kit (Qiagen) according to the manufacturer's instructions. All samples were DNase treated.

### RT-qPCR

cDNA was prepared using the Superscript II First Strand Synthesis Kit (Invitrogen) with Oligo-dT primers, according to the manufacturer's instructions. Relative expression levels were determined using the Roche Universal Probe Library on the Roche Lightcycler 480 after normalization to the geometric mean of three housekeeping genes (α-tubulin, HMBS and TBP) for all experiments except for TRAs, which were normalized to epithelial V-like antigen (EVA) (DeMonte et al., 2007). Technical triplicates were run for all samples and no reverse transcriptase and no template controls were included in all experiments. The primers used for RT-qPCR are listed in supplementary material Table S2.

### TREC

Signal joint T cell receptor delta (TCRD) excision circle (sjTREC) quantification was based on a previously described method (Sempowski et al., 2002). Genomic DNA was isolated from splenocytes by cell lysis (10,000 cells/μl) in 10 mM Tris with 0.05% Tween-20, 0.05% NP-40 and 100 μg/ml Proteinase K. TREC numbers were quantified on a Roche Lightcycler 480 using the following primer/probe set: forward primer, 5′-CATTGCCTTTGAAC-CAAGCTGA-3′; reverse primer, 5′-TTATGCACAGGGTGCAGGTG-3′; probe, FAM-GCAGGTTTTTGTAAAGGTGCTCACTTCT-BHQ (Sigma).

Samples were assayed at 25,000 cell equivalents per PCR reaction. A TREC standard was generated by PCR-cloning a 315-bp fragment, spanning the TCRD single joint site, from thymocyte genomic DNA into a TOPO-TA vector (Invitrogen) (primers: 5′-TAGGGAAGATGGGCCTCTCTG-3′, 5′-GTGTGTCCTCAGCCTTGATCCATC-3′). Serial dilutions of the TREC standard (10^7^, 10^6^, 10^5^, 10^4^, 10^3^, 10^2^ and 10^1^ molecules per reaction) were used to generate a standard curve. To test for equivalent genomic DNA input, we separately amplified the α-tubulin reference gene.

### Western blotting

Nuclear and cytoplasmic protein fractions were prepared using a nuclear extract kit (Active Motif) according to the manufacturer's instructions and processed for western blotting as described (Depreter et al., 2008; Nowell et al., 2011).

### Statistical analysis

Statistical analysis was performed using the one-way ANOVA test (two tailed), as appropriate for normally distributed data (normal distribution was tested using χ^2^ goodness of fit). The alpha level is taken as 0.05. Errors shown are standard deviations throughout. Sample sizes of at least *n*=3 were used for statistical analyses.

## Supplementary Material

Supplementary Material
