# Supplementary material for: Regeneration of the aged thymus by a single transcription factor
Source: Development. 2014 Apr;141(8):1627–37. doi: 10.1242/dev.103614 (PMC3978836; doi:10.1242/dev.103614)
Supplement: Supplementary Material [file supp_141_8_1627__index.html]

Supplementary Material 

# Regeneration of the aged thymus by a single transcription factor

## DEV103614 Supplementary Material

**Files in this Data Supplement:**

- **Supplementary Material**
