# Supplementary material for: Regeneration of the aged thymus by a single transcription factor
Source: Development. 2014 Apr;141(8):1627–37. doi: 10.1242/dev.103614 (PMC3978836; doi:10.1242/dev.103614)
Supplement: Supplementary Material [file supp_141.8.1627_DEV103614.pdf]

Supplementary Figures

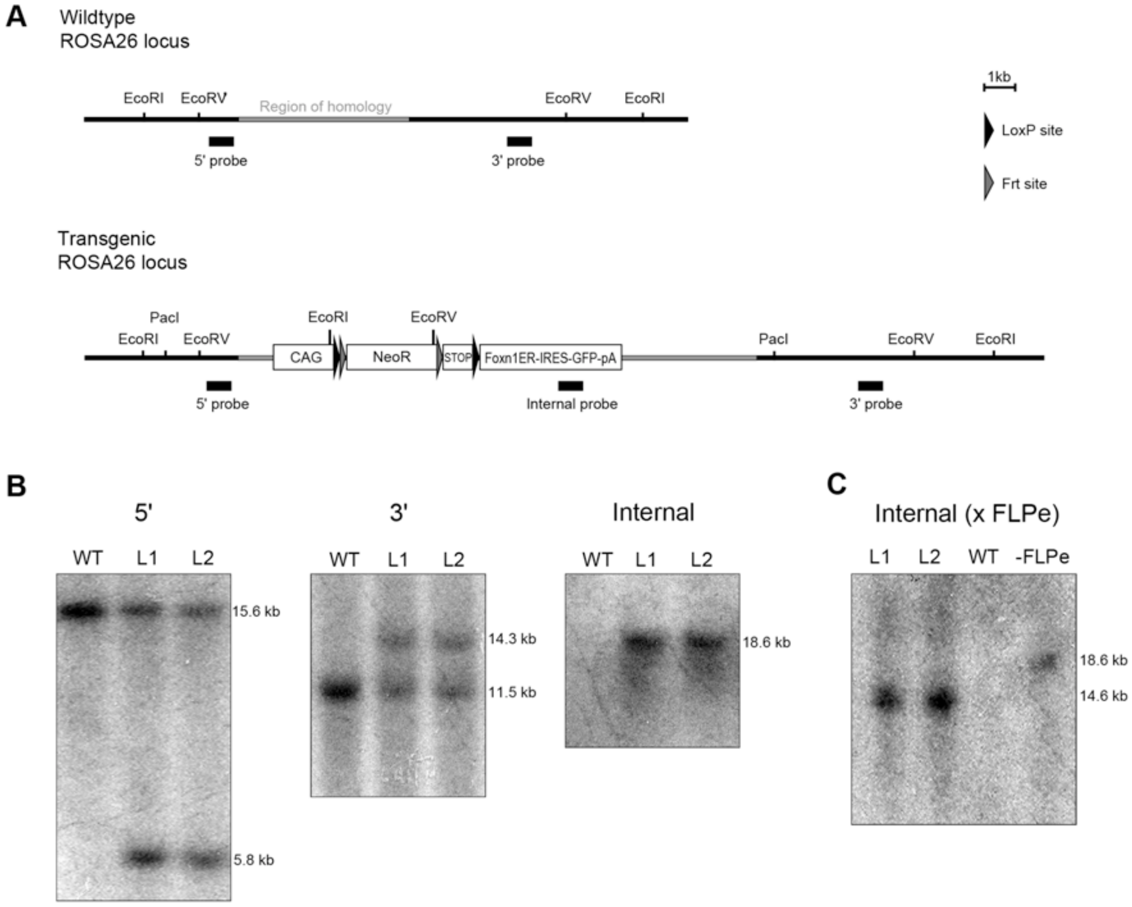

Figure S1. Generation of transgenic *Rosa26*$^{CAG\text{-}STOP\text{-}Foxn1ERT2\text{-}IRES\text{-}GFP}$ mice. (A) The CAG-STOP-Foxn1ERT2 cassette shown in Figure 1 was introduced into the *Rosa26* locus (Soriano, 1999) in mouse E14tg2a ES cells by homologous recombination using standard procedures, generating the *Rosa26*$^{CAG\text{-}NeoR\text{-}STOP\text{-}Foxn1ERT2\text{-}IRES\text{-}GFP}$ allele. This cassette contained a cDNA encoding FOXN1ER^{T2}, under control of the CAG compound promoter and downstream of a LoxP-flanked CMAZ stop cassette (Ashfield et al., 1994), plus an IRES-GFP component to permit monitoring of *Foxn1ERT2* expression. Neomycin-resistant colonies were picked and screened for targeted insertion by Southern blotting, using the strategy shown in (A) above. The position of restriction enzyme sites and Southern blot

hybridization probes are shown for the wild type and transgenic *Rosa26* locus. EcoRI, EcoRV and PacI restriction enzyme digests were used for 5′, 3′ and internal Southern blot analyses, respectively. **(B)** Correctly targeted colonies were identified (B) and used to generate chimeric mice via blastocyst injection. Germline transmission was confirmed from two independent ES cell lines (L1 and L2). **(C)** Founders from each of these *Rosa26*$^{CAG\text{-}NeoR\text{-}STOP\text{-}Foxn1ERT2\text{-}IRES\text{-}GFP}$ lines were crossed with *Tg(CAG-FLPe)* mice (Wallace et al., 2007), in order to remove the neomycin resistance cassette (NeoR). Removal of this cassette was confirmed by Southern blotting**.** These lines were then backcrossed to C57BL/6 mice for three generations before analysis.

```
   1 - ATGGTGTCGCTACTCCCTCCGCAGTCTGACGTCACACTTCCAGGCTCCACCCGACTGGAGGGCGAACCCCAAGGGGACCTCATGCAGGCTCCGGGCCTCCCAGACTCCCCTGCCCCACAG - 120
   1 - M V S L L P P Q S D V T L P G S T R L E G E P Q G D L M Q A P G L P D S P A P Q - 40

 121 - AACAAGCATGCTAACTTCAGCTGCTCGTCGTTTGTGCCTGACGGCCCTCCAGAGAGGACACCCTCACTGCCCCCACACAGCCCCAGCATCGCATCTCCAGACCCAGAGCAGATCCAGGGC - 240
  41 - N K H A N F S C S S F V P D G P P E R T P S L P P H S P S I A S P D P E Q I Q G - 80

 241 - CACTGCACAGCCGGACCCGGCCCGGGCTCCTTCCGCCTTTCTCCTTCAGAAAAGTATCCTGGCTTTGGCTTTGAGGAGGGCCCAGCAGGCAGCCCAGGGCGCTTCCTCAAGGGCAACCAC - 360
  81 - H C T A G P G P G S F R L S P S E K Y P G F G F E E G P A G S P G R F L K G N H - 120

 361 - ATGCCTTTCCACCCCTACAAGAGGCACTTCCATGAGGACATCTTCTCTGAGGCCCAGACGGCCATGGCACTTGATGGACACTCCTTTAAGACTCAGGGGGCACTGGAAGCCTTTGAGGAG - 480
 121 - M P F H P Y K R H F H E D I F S E A Q T A M A L D G H S F K T Q G A L E A F E E - 160

 481 - ATCCCTGTGGACATGGGCGATGCTGAGGCCTTCCTGCCTAGCTTCCCAGCAGAGGCTTGGTGCAATAAACTCCCTTACCCCAGCCAGGAACACAACCAAATTCTGCAGGGGTCAGAGGTC - 600
 161 - I P V D M G D A E A F L P S F P A E A W C N K L P Y P S Q E H N Q I L Q G S E V - 200

 601 - AAGGTCAAGCCCCAAGCTCTGGACAGTGGTCCTGGGATGTACTGCTACCAGCCTCCCTTGCAACATATGTACTGTTCTTCTCAGCCTGCCTTCCATCAGTACTCCCCGGGTGGAGGCAGC - 720
 201 - K V K P Q A L D S G P G M Y C Y Q P P L Q H M Y C S S Q P A F H Q Y S P G G G S - 240

 721 - TACCCTGTGCCCTACCTGGGCTCACCTCACTATCCCTATCAGAGGATTGCACCCCAGGCCAACGCCGAAGGTCACCAGCCACTCTTCCCAAAGCCCATCTACTCTTACAGCATCCTCATC - 840
 241 - Y P V P Y L G S P H Y P Y Q R I A P Q A N A E G H Q P L F P K P I Y S Y S I L I - 280

 841 - TTCATGGCCCTTAAGAACAGTAAGACCGGAAGCCTTCCAGTCAGTGAAATCTACAATTTCATGACGGAGCACTTCCCTTACTTCAAGACTGCTCCTGATGGCTGGAAGAATTCTGTTCGC - 960
 281 - F M A L K N S K T G S L P V S E I Y N F M T E H F P Y F K T A P D G W K N S V R - 320

 961 - CATAACCTGTCCCTCAACAAGTGCTTTGAGAAGGTGGAGAATAAATCCGGAAGTTCCTCTCGAAAGGGCTGTCTGTGGGCCCTCAATCCTTCCAAAATCGACAAGATGCAGGAAGAACTG - 1080
 321 - H N L S L N K C F E K V E N K S G S S S R K G C L W A L N P S K I D K M Q E E L - 360

1081 - CAGAAGTGGAAGAGGAAAGACCCCATTGCTGTGCGCAAAAGCATGGCCAAACCAGAAGAGCTGGACAGCCTCATTGGAGACAAAAGGGAAAAACTGGGCTCTCCGCTGCTGGGCTGTCCA - 1200
 361 - Q K W K R K D P I A V R K S M A K P E E L D S L I G D K R E K L G S P L L G C P - 400

1201 - CCCCCTGGGCTGGCAGGCCCAGGTCCCATCCGGCCCATGGCACCATCAGCTGGTCTTTCCCAGCCTCTGCACCCAATGCATCCAGCTCCAGGCCCCATGCCTGGCAAGAACCCCCTGCAG - 1320
 401 - P P G L A G P G P I R P M A P S A G L S Q P L H P M H P A P G P M P G K N P L Q - 440

1321 - GACCTACTGGGTGGCCATGCTCCCTCCTGCTATGGGCAGACCTACCCACACCTTTCCCCCAGCCTGGCCCCTTCTGGACACCAGCAGCCATTGTTCCCACAGCCAGATGGGCATCTTGAG - 1440
 441 - D L L G G H A P S C Y G Q T Y P H L S P S L A P S G H Q Q P L F P Q P D G H L E - 480

1441 - CTGCAGGCCCAGCCAGGCACCCCCCAGGACTCACCTCTACCTGCCCACACACCACCCAGCCACGGTGCCAAGCTGATGGCTGAGCCTTCCTCAGCCAGGACCATGCACGATACTCTACTG - 1560
 481 - L Q A Q P G T P Q D S P L P A H T P P S H G A K L M A E P S S A R T M H D T L L - 520

1561 - CCAGATGGAGACCTTGGGACTGACCTGGATGCTATCAACCCTTCTCTCACTGACTTCGACTTCCAGGGAAATCTGTGGGAGCAGCTGAAGGATGACAGCTTGGCCCTGGACCCCCTCGTA - 1680
 521 - P D G D L G T D L D A I N P S L T D F D F Q G N L W E Q L K D D S L A L D P L V - 560

1681 - TTGGTGACCTCGTCGCCGACGTCATCCTCCATGTTGCCACCCCCACCAGCAGCCCATTGCTTCCCCCCAGGGCCTTGTCTGGCAGAAACAGGCAATGAGGCAGGTGAACTGGCACCTCCA - 1800
 561 - L V T S S P T S S S M L P P P P A A H C F P P G P C L A E T G N E A G E L A P P - 600

1801 - GGCAGCGGCGGCTCCGGTGCTCTGGGAGACATGCACCTCAGCACTCTCTACTCCGCCTTTGTGGAACTGGAGTCCACGCCCTCCTCAGCAGCTGCCGGCCCTGCCGTGTACCTCAGTCCC - 1920
 601 - G S G G S G A L G D M H L S T L Y S A F V E L E S T P S S A A A G P A V Y L S P - 640

1921 - GGCTCAAAGCCATTGGCTCTGGCTGGAGGTGCTGGATCCGGAGACCCTTCTGCTGGAGACATGAGAGCTGCCAACCTTTGGCCAAGCCCGCTCATGATCAAACGCTCTAAGAAGAACAGC - 2040
 641 - G S K P L A L A G G A G S G D P S A G D M R A A N L W P S P L M I K R S K K N S - 680

2041 - CTGGCCTTGTCCCTGACGGCCGACCAGATGGTCAGTGCCTTGTTGGATGCTGAGCCCCCCATACTCTATTCCGAGTATGATCCTACCAGACCCTTCAGTGAAGCTTCGATGATGGGCTTA - 2160
 681 - L A L S L T A D Q M V S A L L D A E P P I L Y S E Y D P T R P F S E A S M M G L - 720

2161 - CTGACCAACCTGGCAGACAGGGAGCTGGTTCACATGATCAACTGGGCGAAGAGGGTGCCAGGCTTTGTGGATTTGACCCTCCATGATCAGGTCCACCTTCTAGAATGTGCCTGGCTAGAG - 2280
 721 - L T N L A D R E L V H M I N W A K R V P G F V D L T L H D Q V H L L E C A W L E - 760

2281 - ATCCTGATGATTGGTCTCGTCTGGCGCTCCATGGAGCACCCAGTGAAGCTACTGTTTGCTCCTAACTTGCTCTTGGACAGGAACCAGGGAAAATGTGTAGAGGGCATGGTGGAGATCTTC - 2400
 761 - I L M I G L V W R S M E H P V K L L F A P N L L L D R N Q G K C V E G M V E I F - 800

2401 - GACATGCTGCTGGCTACATCATCTCGGTTCCGCATGATGAATCTGCAGGGAGAGGAGTTTGTGTGCCTCAAATCTATTATTTTGCTTAATTCTGGAGTGTACACATTTCTGTCCAGCACC - 2520
 801 - D M L L A T S S R F R M M N L Q G E E F V C L K S I I L L N S G V Y T F L S S T - 840

2521 - CTGAAGTCTCTGGAAGAGAAGGACCATATCCACCGAGTCCTGGACAAGATCACAGACACTTTGATCCACCTGATGGCCAAGGCAGGCCTGACCCTGCAGCAGCAGCACCAGCGGCTGGCC - 2640
 841 - L K S L E E K D H I H R V L D K I T D T L I H L M A K A G L T L Q Q Q H Q R L A - 880

2641 - CAGCTCCTCCTCATCCTCTCCCACATCAGGCACATGAGTAACAAAAGAATGGAGCATCTGTACAGCATGAAGTGCAAGAACGTGGTGCCCCTCTATGACCTGCTGCTGGAGGCGGCGGAC - 2760
 881 - Q L L L I L S H I R H M S N K R M E H L Y S M K C K N V V P L Y D L L L E A A D - 920

2761 - GCCCACCGCCTACATGCGCCCACTAGCCGTGGAGGGGCATCCGTGGAGGAGACGGACCAAAGCCACTTGGCCACTGCGGGCTCTACTTCATCGCATTCCTTGCAAAAGTATTACATCACG - 2880
 921 - A H R L H A P T S R G G A S V E E T D Q S H L A T A G S T S S H S L Q K Y Y I T - 960

2881 - GGGGAGGCAGAGGGTTTCCCTGCCACAGCTTGA - 2913
 961 - G E A E G F P A T A * - 970
```

Figure S2. Sequence of the FOXN1ER^{T2} fusion protein.

The full length mouse *Foxn1* cDNA (red) was fused in-frame to cDNA encoding a mutated ligand binding domain of the human estrogen receptor (ER^{T2}, blue) (Feil et al., 1997). The fusion gene was constructed such that a glycine-rich linker (green) (Zeisig et al., 2004) separates the FOXN1 and ER^{T2} protein domains in the resulting FOXN1ER^{T2} fusion protein. This flexible linker was fused in frame to the sequence encoding the C-terminus of full-length FOXN1, without loss of any FOXN1 amino acids.

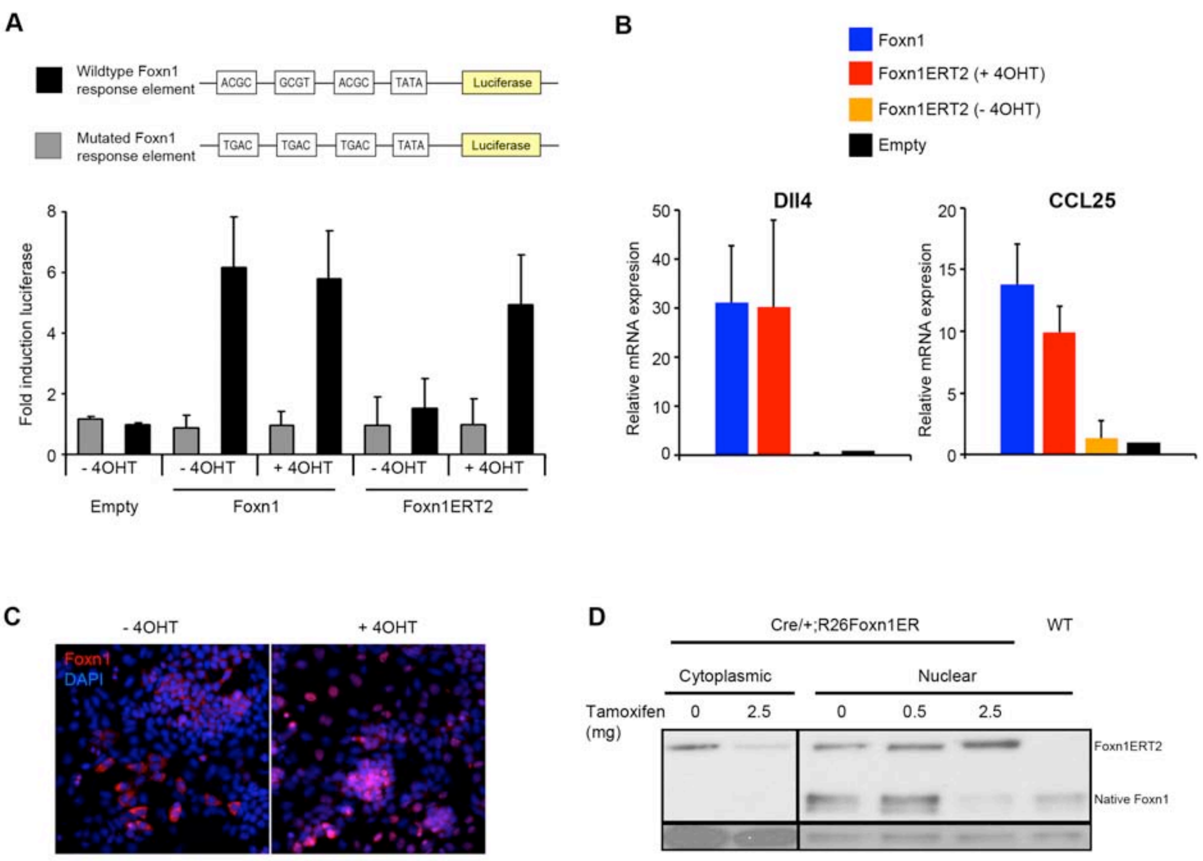

Figure S3. Verification of the transcriptional activity and tamoxifen inducibility of the Foxn1ERT2 fusion protein. (A) The FOXN1ER^{T2} fusion protein is transcriptionally active. Wild-type and mutated FOXN1 transcriptional response elements (Schlake et al., 1997) upstream of a luciferase reporter (Janes et al., 2004) were stably transfected into COS-7 cells. Empty, Foxn1 or Foxn1ERT2 vectors (all driven by the CAG compound promoter (Chambers et al., 2003)) were transfected into these cells and luciferase expression assays were performed after 24 hours. In the presence of 4-hydroxytamoxifen (4OHT), FOXN1 and FOXN1ER^{T2} induced luciferase at a similar level for the wild type response element (black), indicating a comparable transcription activity. FOXN1ER^{T2} was also regulatable by 4OHT. Data represent 2 biological repeats. **(B)** *CAG-Foxn1* and *CAG-Foxn1ERT2* vectors were transiently transfected into E14Tg2A ES cells which were cultured in the presence or absence of tamoxifen, and RT-qPCR analysis for putative FOXN1 target genes, *Dll4* and *CCL25,* was

subsequently performed. FOXN1ER^{T2} and FOXN1 exhibited comparable abilities to regulate putative target genes, *Delta-like 4 (Dll4)* and *Chemokine (C-C motif) ligand 25 (CCL25)* (Bajoghli et al., 2009; Nowell et al., 2011) upon transfection *in vitro*. **(C)** FOXN1ER^{T2} expressed from the *Rosa26$^{CAG\text{-}STOP\text{-}Foxn1ERT2\text{-}IRES\text{-}GFP}$* allele is responsive to tamoxifen *in vitro*. *Rosa26$^{CAG\text{-}STOP\text{-}Foxn1ERT2\text{-}IRES\text{-}GFP}$* ES cells were transfected with a plasmid expressing Cre recombinase in order to excise the Stop cassette, then cultured in the absence and presence of 1μM 4-hydroxytamoxifen (4OHT) and subjected to immunohistochemical analysis of FOXN1 expression using an α-FOXN1 antibody. Panels show cytoplasmic and nuclear Foxn1 expression (red) in the absence (left image) and presence (right image) of 4OHT, respectively. DAPI, blue. **(D)** FOXN1ER^{T2} expressed from the *Rosa26$^{CAG\text{-}STOP\text{-}Foxn1ERT2\text{-}IRES\text{-}GFP}$* allele is responsive to tamoxifen *in vivo.* Image shows immunoblots of sub-cellular protein extracts (Nowell et al., 2011) from E14.5 TECs, following a single tamoxifen (Tam) injection of the amount shown at E13.5, after probing with α-FOXN1 or α-alpha-tubulin; top band is FOXN1ER^{T2}, bottom band is endogenous (native) FOXN1. Bottom panel, loading control (alpha-tubulin). ImageJ quantification of nuclear FOXN1ER^{T2} [arbitrary units] - 0mg tamoxifen, 1U; 0.5mg, 1.5U; 2.5mg, 3.75U. These data demonstrate increased nuclear FOXN1ER^{T2} protein following tamoxifen treatment, with a corresponding decrease in cytoplasmic FOXN1ER^{T2}. Of note is that endogenous FOXN1 protein levels diminished upon translocation of FOXN1ER^{T2} into the nucleus, suggesting the existence of a negative autoregulatory mechanism in fetal TEC.

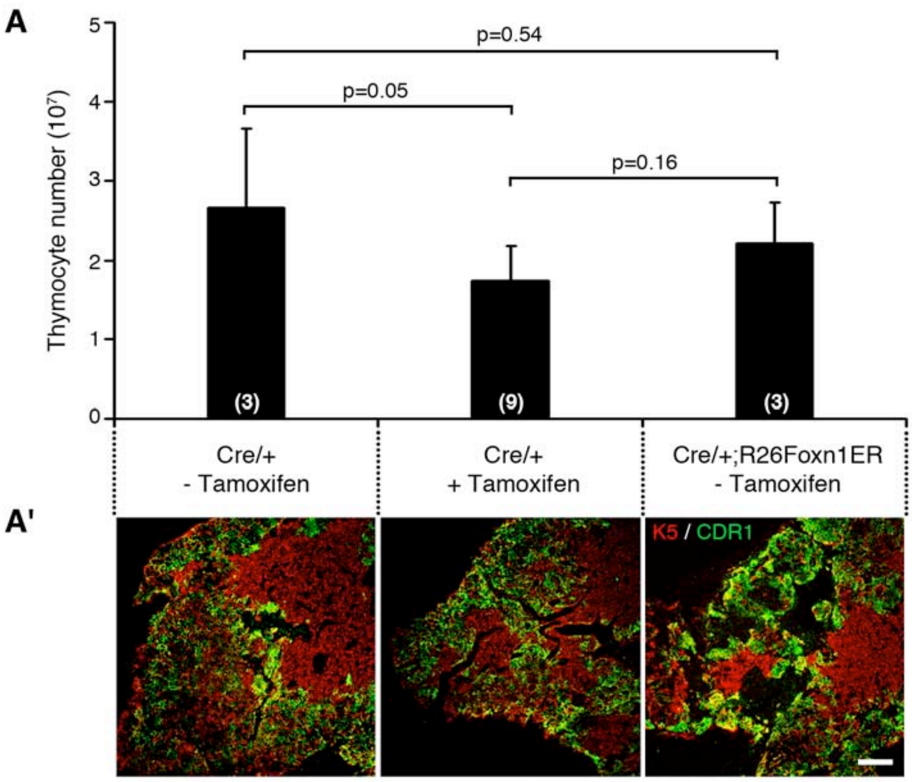

Figure S4. Analysis of thymi from control mice. (A) Total thymocyte number in thymi from 12 month old Cre/+ mice in the presence and absence of tamoxifen treatment, and Cre/+;R26Foxn1ER mice in the absence of tamoxifen treatment. (**A′**) Representative epithelial architecture for thymi from each of these conditions. Cytokeratin 5, mTEC; CDR1, cTEC. No difference in thymic architecture was observed between the different conditions. Scale bar, 100µm.

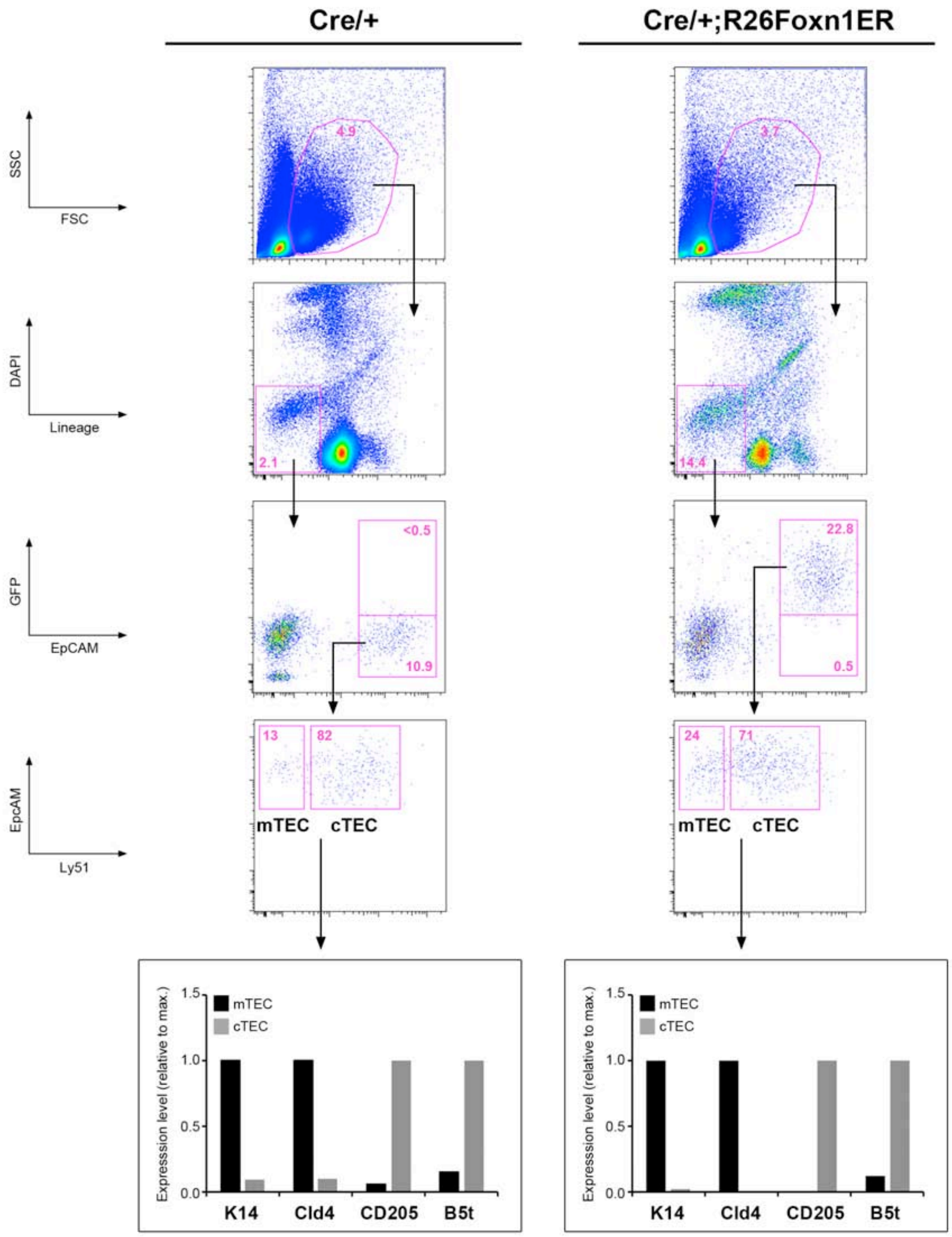

Figure S5. Strategy for flow cytometric isolation of mTEC and cTEC populations.

Representative plots show the gating strategy for isolation of mTEC and cTEC populations from aged Cre/+ and Cre/+;R26Foxn1ER thymi (n > 10). Note that Cre/+;R26Foxn1ER TEC are GFP^+ (expressed from the *Foxn1ER-IRES-GFP* biscistronic mRNA). The flow cytometric

isolation of TEC subsets was verified by gene expression analysis of mTEC and cTEC specific genes in the isolated populations (mTEC, K14 and Cld4; cTEC, CD205 and β5t).

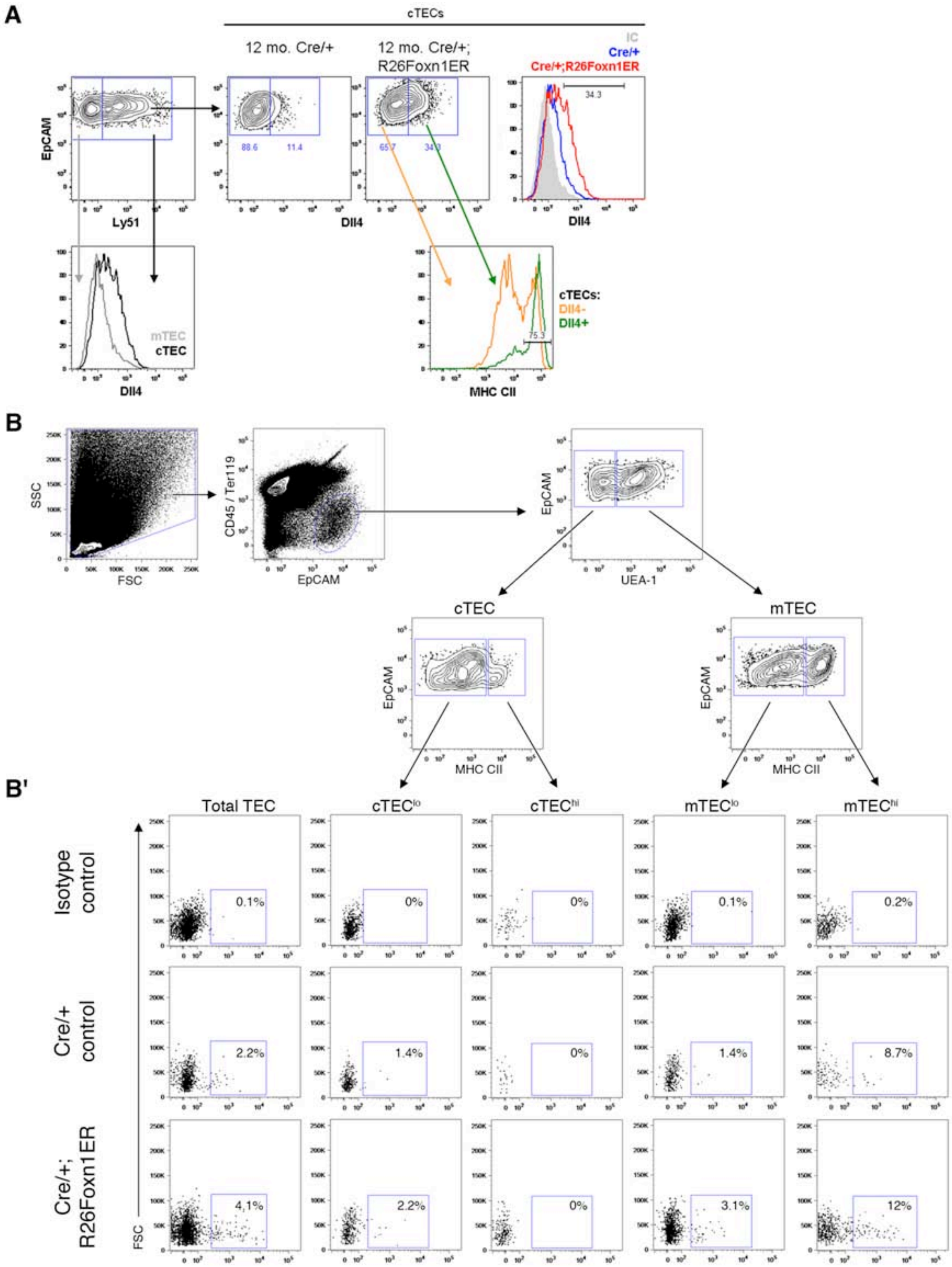

Figure S6. Analysis of Dll4 expression and proliferation in tamoxifen-treated R26Foxn1ER and control mice. (**A**) Flow cytometric analysis of Dll4 expression following induction of FOXN1 expression in aged thymi. Dll4 protein was detected only in cTEC and was predominantly present in MHC Class II^{hi} cells. IC, isotype control. **(B)** Effect of FOXN1

up-regulation on TEC proliferation. Flow cytometric analysis of Ki67 following induction of Foxn1 expression in 12 month-old thymi. Representative plots in (B) show the flow cytometric gating strategy used to resolve the major TEC subpopulations. Total TECs were identified in unenriched thymic cell preparations by gating against $CD45^+$ and $Ter119^+$ and for $EpCAM^+$ cells. Next, four major TEC subpopulations were identified: $cTEC^{lo}$ ($UEA1^-$ MHC CII^{lo}), $cTEC^{hi}$ ($UEA1^-$ MHC CII^{hi}), $mTEC^{lo}$ ($UEA1^+$ MHC CII^{lo}) and $mTEC^{hi}$ ($UEA1^+$ MHC CII^{hi}). (B′) Representative plots show Ki67 expression in these TEC subpopulations. All sample gates based on subpopulation-specific isotype control gates, as indicated.

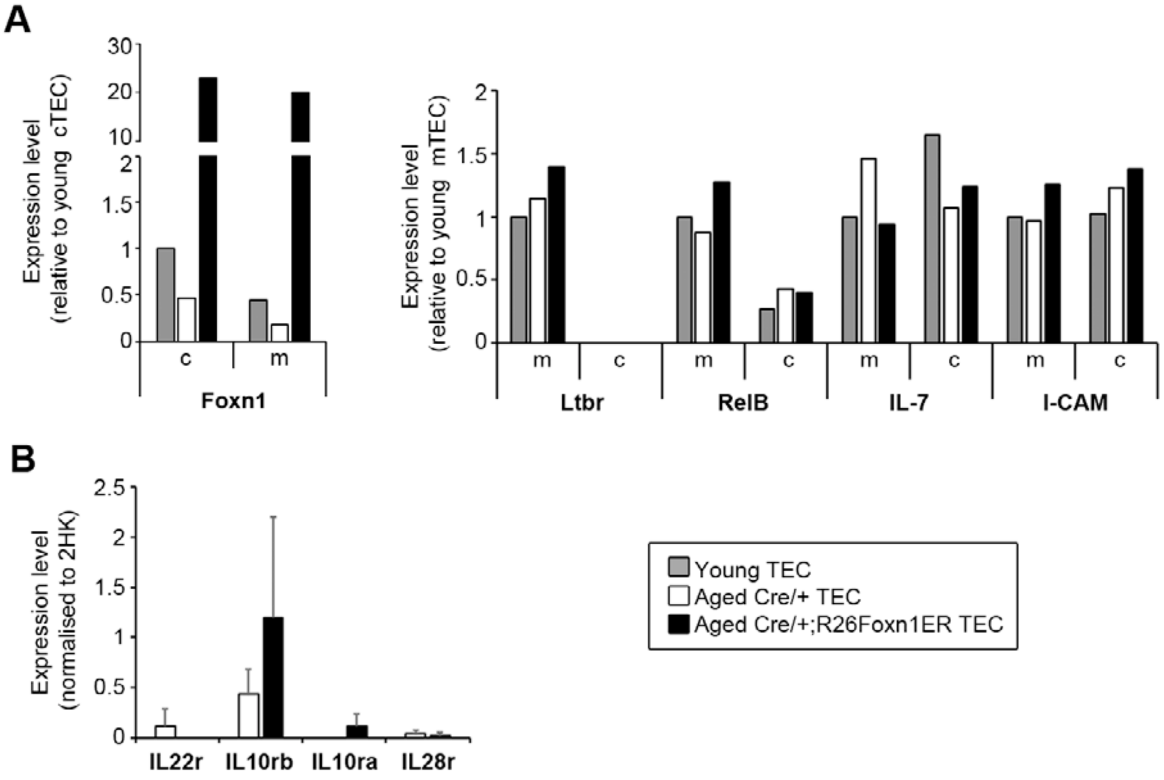

Figure S7. Analysis of gene expression in tamoxifen-treated R26Foxn1ER and control mice. (A) RT-qPCR analysis for genes in TEC isolated from tamoxifen-treated young (3 month old) Cre/+ mice and tamoxifen-treated aged (12-18 month old) Cre/+ and Cre/+;R26Foxn1ER mice. **(B)** RT-qPCR analysis of receptors for the interleukin 10 family of cytokines (Trivella et al., 2010; Wolk et al., 2010) in TEC isolated from 18-24 month old tamoxifen-treated Cre/+ and Cre/+;R26Foxn1ER mice. IL22R and IL10Rb form the receptor for IL20, IL22 and IL24. IL10Rb together with IL10Ra forms the receptor for IL10, and IL10Rb with IL28R forms the receptor for IL28A, IL28B, and IL29. mRNA expression is shown relative to the geometric mean of three housekeeper genes. We could not detect *IL22r* expression in tamoxifen-treated Cre/+;R26Foxn1ER TEC in aged mice indicating that IL22R signaling is not required for Foxn1-mediated thymus regeneration (n=3). c, cTEC; m, mTEC.

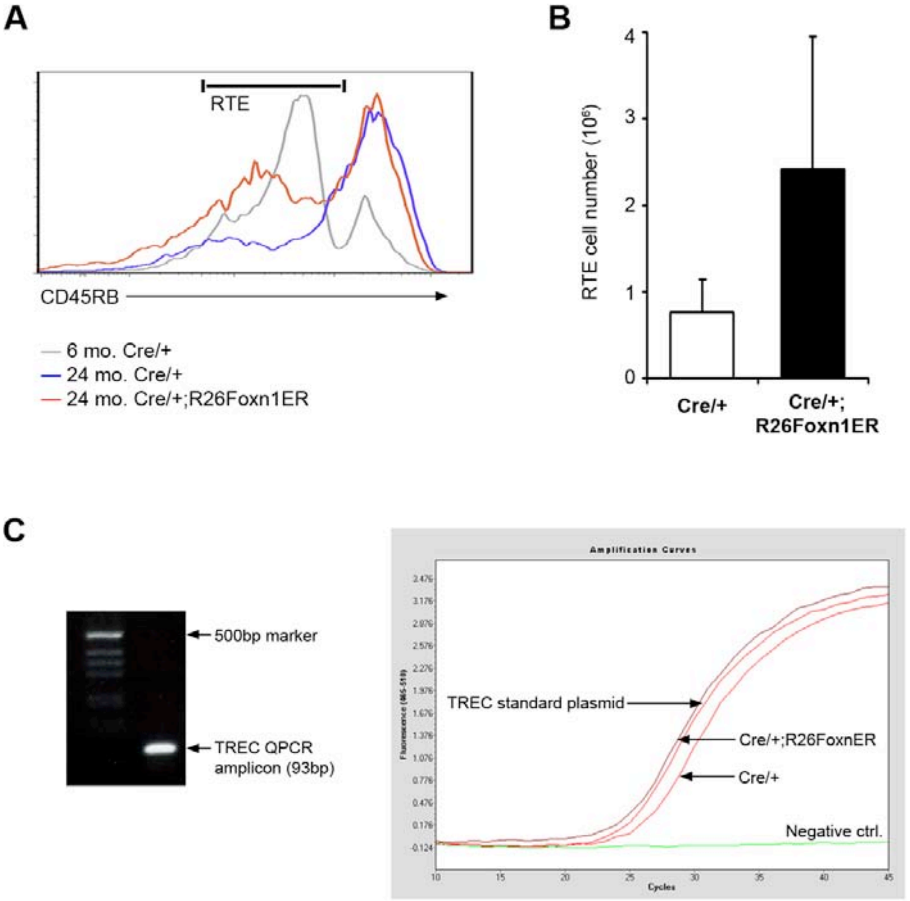

Figure S8. Analysis of recent thymic emigrants in tamoxifen-treated R26Foxn1ER and control mice. (A) Up-regulation of FOXN1 in the aged thymus results in increased numbers of peripheral naïve T cells. Histogram in shows CD45RB profile of $CD4^+$ splenocytes after gating on $CD3^+CD4^+CD62L^+$ cells. RTE are $CD45RB^{lo}$. The proportion of RTE was increased following tamoxifen-treatment of Cre/+;R26Foxn1ER mice (red line). **(B)** Graph shows numbers of RTE as defined in (A) in tamoxifen-treated Cre/+;R26Foxn1ER mice and tamoxifen-treated Cre/+ controls. n=4 for Cre/+ and n=5 for Cre/+;R26Foxn1ER, P=0.075. (**C)** Image shows PCR products from TREC analysis after separation on a 2% agarose gel, and representative graph of amplification curves from qPCR TREC analysis.

Supplementary Tables

Table S1. Antibodies used for IHC and flow cytometry.

**Antibody**	**Clone**	**Supplier**	**Staining**
Aire (D-17)	Polyclonal	SCBT	IHC
B220 PE-Cy7	RA3-6B2	BioLegend	FC – TN/Intrathymic B cell analysis
CD11b FITC	M1/70	E-Bioscience	FC – TN analysis
CD11c FITC	N418	E-Bioscience	FC – TN analysis
CD11c PerCP-Cy5.5	N418	E-Bioscience	FC – TEC isolation
CD25 PerCP-Cy5.5	PC61	BioLegend	FC – TN analysis
CD31 PerCP-Cy5.5	390	BioLegend	FC – TEC isolation
CD3ε FITC	145-2C11	E-Bioscience	FC – TN analysis
CD3ε PE	145-2C11	BioLegend	FC – Splenocyte analysis/isolation
CD4 FITC	RM4-5	BD Biosciences	FC – TN/CD4 v. CD8 analysis, Splenocyte analysis/isolation
CD4 PerCP-Cy5.5	RM4-5	E-Bioscience	FC – TEC isolation
CD44 APC	IM7	E-Bioscience	FC – TN analysis
CD44 APC-eFluor780	IM7	E-Bioscience	FC – Splenocyte analysis/isolation
CD45 APC	30-F11	E-Bioscience	FC – CD4 v. CD8 analysis
CD45 PerCP-Cy5.5	30-F11	E-Biosciences	FC – TEC analysis/isolation
CD45RB APC	C363.16A	E-Bioscience	FC – Splenocyte analysis/isolation

CD62L PE-Cy7	MEL-14	E-Bioscience	FC – Splenocyte analysis/isolation
CD8 PerCP-Cy5.5	53-6.7	E-Bioscience	FC – Splenocyte analysis/isolation
CD8α FITC	53-6.7	E-Bioscience	FC – TN analysis
CD8α PE	53-6.7	BD Biosciences	FC – CD4 v. CD8 analysis
CD8α PerCP-Cy5.5	53-6.7	E-Bioscience	FC – TEC isolation
CDR1	CDR1	Gift from B Kyewski	IHC
c-kit APC-eFluor780	2B8	E-Bioscience	FC – TN analysis
Cytokeratin 14 (AF 64)	Polyclonal	Covance	IHC
Cytokeratin 5 (AF 138)	Polyclonal	Covance	IHC
Cytokeratin 8	Troma1	DSHB	IHC
DAPI (4',6-Diamidino-2-Phenylindole, Dihydrochloride)		Invitrogen	Viability dye
EpCAM APC	G8.8	BioLegend	FC – TEC analysis/isolation
Flt3 PE	A2F10	BioLegend	FC – TN/ETP analysis
Foxn1 (G-20)	Polyclonal	SCBT	IHC, Immunoblotting
Gr-1 FITC	RB6-8C5	E-Bioscience	FC – TN analysis
Ki-67 Isotype control (Mouse IgG1,k PE)	MOPC-21	BD Biosciences	FC – TEC analysis

Ki-67 PE	B56	BD Biosciences	FC – TEC analysis
Ly51 PE	6C3	BioLegend	FC – TEC analysis/isolation
MHC Class II PE-Cy7	M5/114.15.2	BioLegend	FC – TEC analysis/isolation
NK1.1 FITC	PK136	BD Biosciences	FC – TN analysis
Pan-Cytokeratin	Polyclonal	DAKO	IHC
TCRβ FITC	H57-597	BD Biosciences	FC – TN analysis
TCRβ PerCP-Cy5.5	H57-597	BioLegend	FC – TEC isolation
TCRγδ FITC	GL3	BioLegend	FC – TN analysis
Ter119 FITC	Ter119	E-Bioscience	FC – TN analysis
Ter119 PerCP-Cy5.5	Ter119	E-Bioscience	FC – TEC analysis/isolation
UEA-1 biotin	Lectin	Vector Labs	FC – TEC analysis/isolation
ΔNp63 (N-16)	Polyclonal	SCBT	IHC

Table S2. Sequences of primers for RT-qPCR.

Gene	Forward primer (5′-3′)	Reverse primer (5′-3′)
Aire	GGTTCCTCCCCTTCCATC	GGCACACTCATCCTCGTTCT
Alpha-tubulin	CGGACCACTTCAAGGACTAAA	ATTGCCGATCTGGACACC
CCL25	GAGTGCCACCCTAGGTCATC	CCAGCTGGTGCTTACTCTGA
CD40	GAGTCAGACTAATGTCATCTGTGGTT	ACCCCGAAAATGGTGATG
CD80	TCGTCTTTCACAAGTGTCTTCAG	TTGCCAGTAGATTCGGTCTTC
Csna	GACATCTCTCAGGAACTCCACA	TCCATAGAATGAATAGAGAGACATGAG
Csnb	GGTGAATCTCATGGGACAGC	TGACTGGATGCTGGAGTGAA
CtsL	CAAATAAGAATAAATATTGGCTTGTCA	TCGTCTTTCACAAGTGTCTTCAG
Cxcl12	GGTTCTTCGAGAGCCACATC	TGTTCTTCAGCCGTGCAA
Cyclin D1	GAGATTGTGCCATCCATGC	CTCCTCTTCGCACTTCTGCT
Dll4	AGGTGCCACTTCGGTTACAC	GGGAGAGCAAATGGCTGATA
EVA	TGTGCTTCCACTTCTCCTGA	TCCACAGCTTCTGTAGGACAAA
FgfR2IIIb	CGGGGTGTTGGAGTTCAT	CCTGCGGAGACAGGTAACA
Foxn1	TGACGGAGCACTTCCCTTAC	GACAGGTTATGGCGAACAGAA
Fzd1	ATCTGGTCCGGCAAGACA	GCTGTTGGTAAGCCTCGTGT
Fzd3	GCAGCCTCCACAGGTCAC	ACATGCTGCCGTGAGGTAG
Fzd4	AACTTTCACGCCGCTCAT	CCGAACAAAGGAAGAACTGC
GAD67	ATACAACCTTTGGCTGCATGT	TTCCGGGACATGAGCAGT
HMBS	TCCCTGAAGGATGTGCCTAC	ACAAGGGTTTTCCCGTTTG
I-FABP	ACGGAACGGAGCTCACTG	TTACCAGAAACCTCTCGGACA
Ins2	GAAGTGGAGGACCCACAAGT	AGTGCCAAGGTCTGAAGGTC
Kit L	TCAACATTAGGTCCCGAGAAA	ACTGCTACTGCTGTCATTCCTAAG

Lrp6	TCCTCGAGCTCTGGCACT	CCTCCCCACTCAGTCCAATA
Pax1	CTCCGCACATTCAGTCAGC	TCTTCCATCTTGGGGGAGTA
Spt1	TGAAACTTCTGGAACTGCTGA	TTTTGATCAGTACTATCTGCCTGAG
TBP	GGGGAGCTGTGATGTGAAGT	CCAGGAAATAATTCTGGCTCA
Tff3	CTGGGATAGCTGCAGATTACG	CATTTGCCGGCACCATAC
Wnt10a	GGCGCTCCTGTTCTTCCTA	GTCGTTGGGTGCTGACCT
Wnt11	CAGGATCCCAAGCCAATAAA	TCCAGGGAGGCACGTAGA
Wnt4	ACTGGACTCCCTCCCTGTCT	TGCCCTTGTCACTGCAAA
Wnt5b	AGCACCGTGGACAACACAT	AAGGCAGTCTCTCGGCTACC
Wnt7a	CGCTGGGAGAGCGTACTG	CGATAATCGCATAGGTGAAGG
Wnta3a	CTTAGTGCTCTGCAGCCTGA	GAGTGCTCAGAGAGGAGTACTGG

References

Ashfield, R., Patel, A. J., Bossone, S. A., Brown, H., Campbell, R. D., Marcu, K. B. and Proudfoot, N. J. (1994) 'MAZ-dependent termination between closely spaced human complement genes', *EMBO J* 13(23): 5656-67.

Bajoghli, B., Aghaallaei, N., Hess, I., Rode, I., Netuschil, N., Tay, B. H., Venkatesh, B., Yu, J. K., Kaltenbach, S. L., Holland, N. D. et al. (2009) 'Evolution of genetic networks underlying the emergence of thymopoiesis in vertebrates', *Cell* 138(1): 186-97.

Chambers, I., Colby, D., Robertson, M., Nichols, J., Lee, S., Tweedie, S. and Smith, A. (2003) 'Functional expression cloning of Nanog, a pluripotency sustaining factor in embryonic stem cells', *Cell* 113(5): 643-55.

Feil, R., Wagner, J., Metzger, D. and Chambon, P. (1997) 'Regulation of Cre recombinase activity by mutated estrogen receptor ligand-binding domains', *Biochem Biophys Res Commun* 237(3): 752-7.

Janes, S. M., Ofstad, T. A., Campbell, D. H., Watt, F. M. and Prowse, D. M. (2004) 'Transient activation of FOXN1 in keratinocytes induces a transcriptional programme that promotes terminal differentiation: contrasting roles of FOXN1 and Akt', *J Cell Sci* 117(Pt 18): 4157-68.

Nowell, C. S., Bredenkamp, N., Tetelin, S., Jin, X., Tischner, C., Vaidya, H., Sheridan, J. M., Stenhouse, F. H., Heussen, R., Smith, A. J. et al. (2011) 'Foxn1 regulates lineage progression in cortical and medullary thymic epithelial cells but is dispensable for medullary sublineage divergence', *PLoS Genet* 7(11): e1002348.

Schlake, T., Schorpp, M., Nehls, M. and Boehm, T. (1997) 'The nude gene encodes a sequence-specific DNA binding protein with homologs in organisms that lack an anticipatory immune system', *Proc Natl Acad Sci U S A* 94(8): 3842-7.

Soriano, P. (1999) 'Generalized lacZ expression with the ROSA26 Cre reporter strain', *Nat Genet* 21(1): 70-1.

Trivella, D. B., Ferreira-Junior, J. R., Dumoutier, L., Renauld, J. C. and Polikarpov, I. (2010) 'Structure and function of interleukin-22 and other members of the interleukin-10 family', *Cell Mol Life Sci* 67(17): 2909-35.

Wallace, H. A., Marques-Kranc, F., Richardson, M., Luna-Crespo, F., Sharpe, J. A., Hughes, J., Wood, W. G., Higgs, D. R. and Smith, A. J. (2007) 'Manipulating the mouse genome to engineer precise functional syntenic replacements with human sequence', *Cell* 128(1): 197-209.

Wolk, K., Witte, E., Witte, K., Warszawska, K. and Sabat, R. (2010) 'Biology of interleukin-22', *Semin Immunopathol* 32(1): 17-31.

Zeisig, B. B., Milne, T., Garcia-Cuellar, M. P., Schreiner, S., Martin, M. E., Fuchs, U., Borkhardt, A., Chanda, S. K., Walker, J., Soden, R. et al. (2004) 'Hoxa9 and Meis1 are key targets for MLL-ENL-mediated cellular immortalization', *Mol Cell Biol* 24(2): 617-28.
